# Habitat conditions shape the phyllosphere mycobiome of the critically endangered Abies nebrodensis

**DOI:** 10.3389/fpls.2026.1876158

**Published:** 2026-07-24

**Authors:** Giovanni Emiliani, Sara Barberini, Gianni Della Rocca, Francesco Bigazzi, Salvatore Moricca, Rosario Schicchi, Roberto Danti

**Affiliations:** 1 National Research Council of Italy, Institute for Sustainable Plant Protection (CNR-IPSP), Sesto, Fiorentino, Italy; 2 Department of Agricultural, Food, Environmental and Forestry Science and Technology (DAGRI), Plant Pathology and Entomology Section, University of Florence, Florence, Italy; 3 Agricultural, Food and Forest Department, University of Palermo, Palermo, Italy

**Keywords:** fungal diversity, microbiome, phyllosphere, relic population, Sicilian fir

## Abstract

The phyllosphere harbors diverse fungal communities that influence tree health, adaptation, and growth. This study investigated the phyllosphere mycobiome of the critically endangered *Abies nebrodensis*, using ITS2 metabarcoding. Samples were collected from 10 trees distributed across three sites during a single sampling campaign. Influence of tissue health state (green vs. blighted twigs) and variation of habitat conditions on diversity and composition of the mycobiome was evaluated. The 4,501 amplicon sequence variants (ASVs) detected were dominated by Ascomycota, particularly Dothideomycetes and Sordariomycetes. The endospheric community showed reduced diversity compared to the full phyllospheric community; however, both were dominated by Valsaceae and Phaeosphaeriaceae and by the genera *Phaeosphaeria* and *Lachnellula*. Tissue health and site conditions significantly affected diversity and composition of the full phyllospheric but not of endospheric communities. Valsaceae were consistently detected within the core mycobiota of Sicilian fir, together with melanized and extremotolerant genera such as *Knufia*, *Perusta*, *Cladosporium*, *Alternaria*, and *Sarcinomyces*. Despite the study was based on a single spring snapshot, our findings indicate habitat variation as a major driver of the phyllosphere mycobiome of *A. nebrodensis*. The occurrence of Valsaceae and extremotolerant fungi in the core mycobiome suggests that these taxa may play a functional role potentially contributing to the holobiont adaptation to a harsh environment.

## 1 Introduction

Climatic and edaphic factors directly affect growth, physiology and health of forest trees (Ali et al., 2020). Because of climate change, high temperatures and drought may lead to water scarcity, reduced biomass production, and, indirectly, increased vulnerability to pathogens (Desprez-Loustau et al., 2007; Sturrock et al., 2011). The impact of climate warming can be particularly alarming for endangered species, especially those with small population sizes (Panetta et al., 2018). Reduced genetic variability in such populations may limit their ability to adapt to new environmental challenges (Jump et al., 2009).

The Sicilian fir, *Abies nebrodensis* (Lojac.) Mattei, is an endemic species found in northern Sicily (Italy), restricted to a small area of 84 hectares within the Madonie Regional Park and protected under EU legislation (Habitat Directive). The current population consists of only 30 relic adult trees and a fluctuating number of naturally regenerating juveniles. The Sicilian fir is classified as “Critically Endangered” under criterion D of the International Union for Conservation of Nature Red List, due to its population being estimated at fewer than 250 mature individuals and is currently listed as CR-D in the Red List of Threatened Species (Thomas, 2017).

The habitat where the remaining Sicilian fir trees grow spans between 1,400 and 1,700 meters above sea level. It extends from the vegetation belt dominated by Quercus petraea (Matt.) Liebl. and Ilex aquifolium L. to areas characterized by Fagus sylvatica L., predominantly on north- to north-west-facing slopes with a quartz-arenitic substrate. The distribution range of A. nebrodensis is extremely fragmented, with trees growing under diverse microclimatic and ecological conditions. Some individuals are completely isolated, often found near ridges, screes, or stony terrain, while others are located near or within beech and oak groves.

Management of this relic population involves a series of conservation measures focused on habitat improvement and the monitoring of plant health status (Frascella et al., 2022). A recent phytosanitary survey (Frascella et al., 2024) reported that most adult trees were in a relatively good state of health, though some exhibited foliar symptoms such as needle reddening, needle cast, and twig blight. These symptoms were attributed to a combination of harsh climatic conditions and opportunistic fungal pathogens from the genera Rhizosphaera and Cytospora (Frascella et al., 2024).

Biodiversity and ecosystem functioning are driven by the interaction between plants and microbial communities (Wardle et al., 2004). Indeed, it is now well established that a host plant, together with its associated microbiota, should be regarded as a holobiont functioning as a single ecological and evolutionary unit (Lemanceau et al., 2017; Zilber-Rosenberg and Rosenberg, 2008). Within the microbiota, fungi are capable of positively influencing plant health and development by providing a wide range of functional metabolic capabilities and preventing infections caused by phytopathogens (Collinge et al., 2022; Sarkar et al., 2025). The vast genetic diversity contained within plant-associated microbiomes is often referred to as an “extended genome” (Turner et al., 2013; Hawkes et al., 2021). This additional genetic reservoir enhances the plant’s ability to respond to and withstand a broader range of environmental stresses and perturbations (Angulo et al., 2022; Petipas et al., 2021). The formation and structuring of the fungal endophytic and epiphytic communities are multistep processes influenced by various pedoclimatic and host-related factors (Trivedi et al., 2020). A plant may acquire its microbiota through horizontal transmission - that is, from the environment (mainly the rhizosphere or phyllosphere) - and through vertical transmission, from one generation to the next via seeds or vegetative propagules (Mishra et al., 2021).

Plants are highly selective in recruiting their microbiota depending on host-specific traits. Plant genotype, developmental stage, compartments, and immune system are considered key determinants in the final assembly of the phytobiome (Coleman-Derr et al., 2016; Dastogeer et al., 2020). The endosphere of roots, stems, leaves, flowers, fruits, and seeds exhibit different biotic and abiotic characteristics. Considering the variation in nutrient and water availability, hormone production, pH, and UV radiation, it can be hypothesized that each microenvironment supports the growth of microorganisms equipped with the necessary metabolic and resistance traits (Cregger et al., 2018; Semenzato et al., 2025). As expected, this leads to the formation of diverse microbial communities in the different host compartments and in the soil, where fungi can either compete for accessible resources or cooperate to form stable, coexisting populations (Dastogeer et al., 2020).

The importance of tree microbiome associations to either environmental adaptation or against pest and pathogens is recognized (Terhonen et al., 2019; Terhonen et al., 2023). Phyllosphere microbiome, though less studied than that of rhizosphere, has received particular attention in the last few years showing a high species richness both in bacteria and in fungal communities (Laforest-Lapointe et al., 2016; Menkis et al., 2015). Among the organisms inhabiting the foliar leaves, one can distinguish between those residing on the leaf surface (epiphytes) and those living in the leaf tissues (endophytes), both playing a crucial role in plant defense. Plant fungal epiphytes may induce resistance to pathogens in plants or may also act as pathogens themselves (Kowalski et al., 2015), while fungal foliar endophytes can protect their host against pest and pathogens (Calhoun et al., 1992; Frasz et al., 2014; McMullin et al., 2018), can increase tree tolerance to abiotic stresses like drought, salinity and heat, and can be involved also in plant growth promotion and in litter decomposition (Jia et al., 2020). On the other hand, endophytic fungi colonizing healthy plant tissues can potentially switch to pathogens or saprotrophs, usually when the plant is under stress (Liao et al., 2025).

According to these findings, the studies of plant mycobiota (i.e., the total fungal community of a plant) using cultivation-based or cultivation-independent methods via high throughput sequencing methods (HTS) have increased within the last decade, describing a plethora of fungal communities involved in plant defense, adaptation, and communication with the surrounding environment. It has been proven that this community changes according to host genotype (Qian et al., 2018, Cordier et al., 2012; Redondo et al., 2022), environmental conditions (Linnakoski et al., 2017), tree health, organ and interactions with other organisms (Kovalchuk et al., 2018).

The aim of this work was to analyze, using an HTS approach, the total phyllosphere and endospheric (i.e., colonizing internal tissues) fungal community of the relic natural population of *A. nebrodensis* trees. The analysis focused on community diversity and composition of phyllospheric fungi in relation to: 1) variation of habitat conditions, comparing trees subdivided into three nuclei located in different edaphic and microclimatic sites, 2) the health state of crown samples, based on symptoms on twigs and needles (e.g., reddening and blight), 3) different plant compartments, e.g., needles and twigs. We hypothesized that fungal communities are subject to changes in composition and structure based on the microclimatic conditions of the habitat and that symptomatic needles and twigs host fungal assemblages enriched with pathogenic and saprophytic fungi.

## 2 Materials and methods

### 2.1 Site description

**Figure 1:**
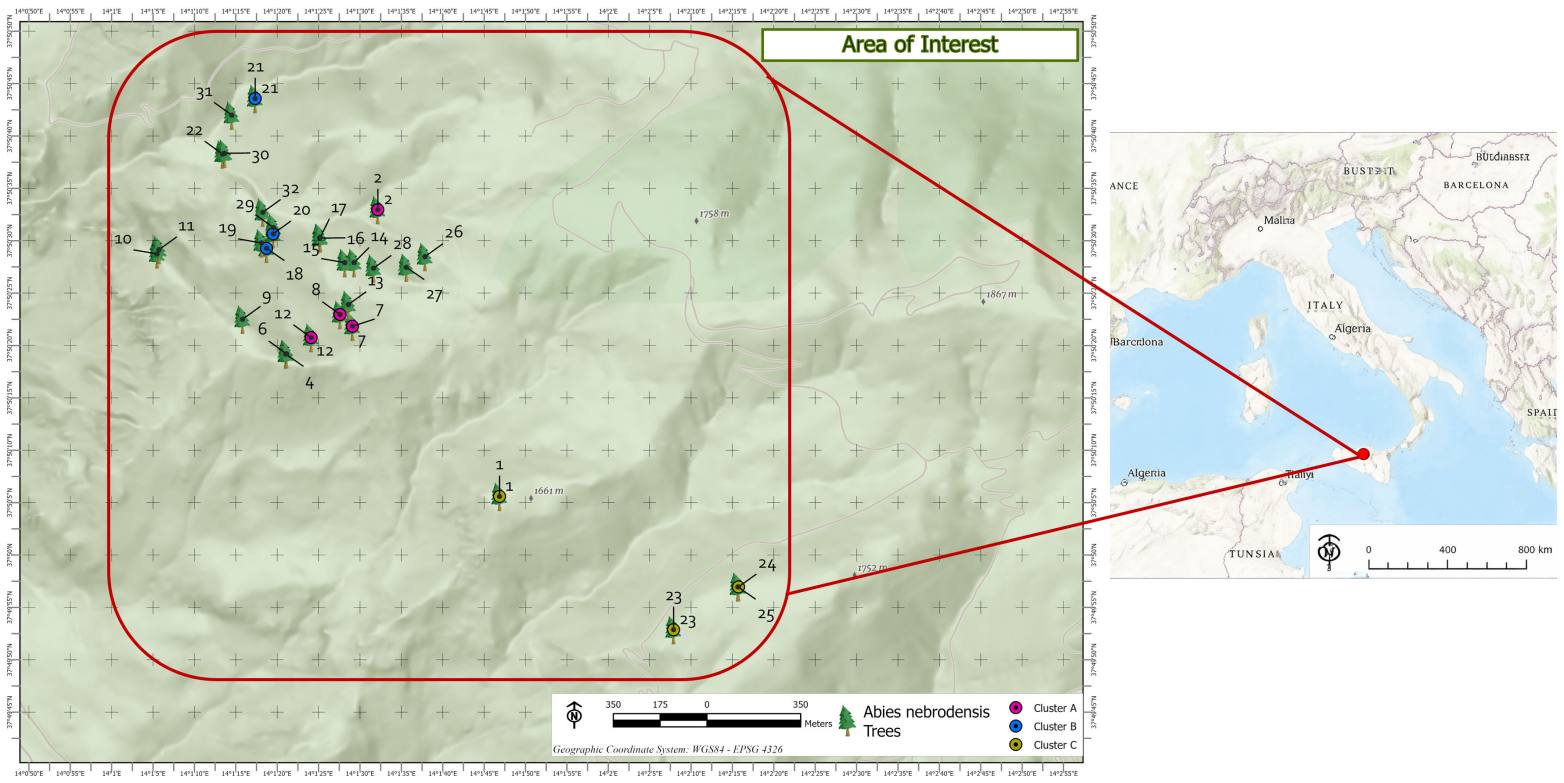
Geographic location of the relict population of *A. nebrodensis* in the Madonie Regional Park (Sicily, Italy) indicated by the red dot. The map shows the distribution of the 30 adult plants grouped in the three sites (A, violet dots; B, blue dots; C, green dots) of the Madonie Regional Park considered in the present study.

A survey of fungal communities was conducted in the relic *A. nebrodensis* population, located in the municipality of Polizzi Generosa (southern Italy) within the Madonie Regional Park (**Figure 1**). The 30 relic trees grow in a restricted area of 84-ha in the Madonie range, between 1400 and 1700 m a.s.l. Among them, 10 individuals located in 3 sites of the habitat differing for soil traits, microclimatic conditions, surrounding vegetation and elevation were sampled. Site A was referred as “mixed rocky slope” (tree ID 2, 7, 8 and 12); site B as “closed-canopy beech forest” (tree ID 18, 20, and 21); site C as “exposed ridge” (tree id. 1, 23, 24). Their description is reported in **Table 1**.

**Table 1:** Main phytopathological features of sampled trees subdivided in three habitat sites, differing for soil traits, microclimate and surrounding vegetation.

Tree n.	Health state	Site	Ecological conditions
7	Crown damaged (3%). Desiccation on the distal part of the branches. Blight and needle reddening on the terminal shoots. Wounds caused by herbivores on lower branches.	A	A: Mixed rocky slope Isolated trees on stony ground, the soil is rocky, shallow, and nutrient poor. Vegetative soil cover is absent or limited to sparse, uneven shrubs and grasses. Extensive bare rock creates harsh microclimatic conditions, particularly in summer, characterized by strong winds, high temperatures, and intense solar radiation
8	Crown damaged (1%). Wounds caused by herbivores on lower branches. Sporadic shoots with blight and needle reddening.	A	
12	Crown damaged (20%). Desiccation and reddening on the distal part of branches and twigs. Chlorosis affecting some of the 1st and 2nd order branches.	A	
2	Crown damaged (5%). Defoliation and drying out of 1st order branches and of the distal part (50cm) of 2nd order branches. Chlorosis and microphyllia on some 1st order twigs.	A	
21	Crown damaged (5%). Desiccations in the distal half of the 2nd order branches. Blight and needle reddening on the distal part on 2 twigs of the 2nd order.	B	B: Closed-canopy beech forest *A. nebrodensis* trees are interspersed in beech grove. Edaphic conditions are characterized by the presence of a soil layer with a greater content of organic substance thanks to the build-up of beech litter. Microclimatic conditions are also mitigated by the presence of the beech canopy shading the lower portions of the *A. nebrodensis* crown as well as by greater relative humidity.
20	Crown missing 50%. Defoliation of the lower half the crown due to shading by the nearby beech trees; the remaining crown is healthy. Rare desiccation on 1st the order branches.	B	
18	Crown missing (20%), defoliation due to shading by the nearby beech trees. Desiccation of 3 branches at a height of one meter from the ground.	B	
1	Crown damaged (15%). Sporadic twig blight and needle reddening (mainly facing North). Wounds and twig desiccation caused by herbivores on the lower branches.	C	C: Exposed ridge Isolated trees, located far from the main nucleus, grow on stony and rocky soils with uneven grass and shrub cover dominated by juniper cushions. These trees are typically found near ridgelines, where they are highly exposed to strong winds and experience particularly harsh climatic conditions.
23	Crown damaged (5%). Needle reddening and small leaves on the 2nd order twigs.	C	
24	Crown damaged (5%). Twigs damaged by herbivores (mechanical wounds).	C	

A descriptive summary of the ecological conditions prevailing in each group is reported.

### 2.2 Leaf sampling and analysis

The sampling took place in May 2021. Three-four adult trees per site (for a total of 10 trees) were selected, choosing individuals with similar crown size (height or diameter). A pair of twigs 20–25 cm long was taken from the distal portion of branches of each sampled tree, per each cardinal point: one green (showing no disorders) and hereinafter defined as “healthy”, and one blighted with brown needles still attached (defined as “symptomatic”). Eight twigs per tree were hence collected, for a total of 80 samples (40 healthy and 40 symptomatic). The sampled twigs were separately placed in plastic bags and transported with a portable cooler to the laboratory where they were maintained at 4 °C and were processed within 3 days. Symptomatic and heathy twigs were separately pooled by each single tree to avoid any potential biases. The pooled samples of each tree, both symptomatic and asymptomatic, were then divided into 2 groups: one was surface-sterilized (SS) and then stored at -80 °C, one was stored directly at -80 °C (NS) in sterile tubes until further processing. Each sample (NS or SS) was then divided into two parts: the leaf part, made up of needles, and the woody twig. SS samples were treated as follow: they were surface sterilized by immersion in a 70% ethanol solution for 1 min and then in a sodium hypochlorite solution (4–5% active chlorine) for 4 min; finally, they were rinsed in sterile distilled water and blotted on sterile filter paper according to the protocol of Frascella et al. (2024) already used for this specie.

**Figure 2:**
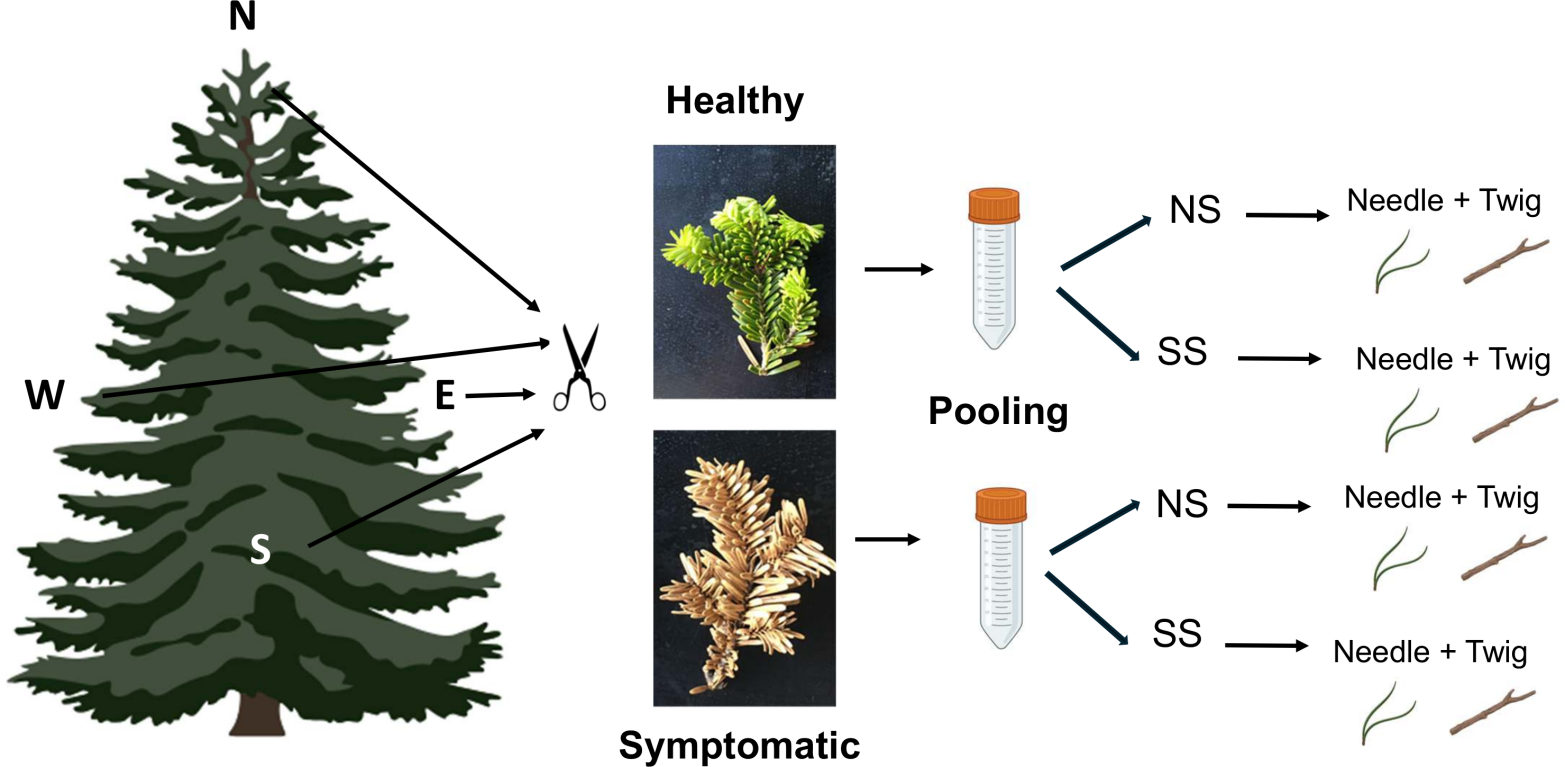
Sampling strategy: For each tree, symptomatic (blighted) and healthy twig samples were collected from the four cardinal directions and separately pooled. For each pool, half were surface-sterilized (SS) while the other half were non-sterilized (NS). Needles and twigs both SS and NS were then separately stored and processed.

Eight pools per tree were hence obtained (80 sample pools in total, **Figure 2**) for subsequent metagenomic analyses.

### 2.3 DNA extraction and rDNA sequencing

Frozen needles and twigs were grinded with liquid nitrogen; 60 mg (fresh weight) of plant material was used for genomic DNA extraction with the Mini Plant II kit (Macherey-Nagel) according to the manufacturer’s instructions. DNA concentrations were determined using Qubit Fluorometer (Thermo Fisher Scientific, USA). The Internal Transcribed Spacer 2 region (ITS2) of the ribosomal operon was amplified using the degenerated primers ITS9/ITS4 pair (ITS9: GAACGCAGCRAAIIGYGA; ITS4: TCCTCCGCTTATTGATATGC; Venice et al., 2021) with Illumina adapters (TCGTCGGCAGCGTCAGATGTGTATAAGAGACAG and GTCTCGTGGGCTCGGAGATGTGTATAAGAGACAG, respectively) using a proofreading Taq DNA polymerase (Platinum II Taq Hot Start DNA polymerase, Invitrogen) with the following protocol: 94 °C 2 min, 35 cycles of 94 °C 30 sec, 58 °C 30 sec, 68 °C 30 sec, final extension 68 °C 5 min. PCR negative controls (no samples added) were used to evaluate that there was no contamination during the preparation for PCR prior to send the samples to the external company. Four PCR reactions were grouped and purified by Wizard SV Gel and PCR clean up kit (Promega Corporation) prior to the library construction. Amplicons’ concentration was measured with a Qubit BR dsDNA assay kit (Thermo Fisher) and showed to be between 0.2 ng/μl and 23.2 ng/μl; amplicons’ integrity was assessed by checking 20 random samples with the Tape Station 4150 (Agilent) capillary electrophoresis. Four samples didn’t satisfy the concentration requirements and were excluded from the analysis.

Amplicons were indexed according to the Illumina Metagenomic protocol using the Nextera XT Index Kit v2 (Illumina). Final library concentrations were measured with the Qubit HS dsDNA assay kit (Thermo Fisher) and used to prepare one 7 nanomolar equimolar pool. The libraries have been sequenced by Genartis SRL (Verona, Italy) using Illumina run MiSeq™ (Caporaso et al., 2012) with a paired-end strategy (2 × 300 bp, NexteraXT index kit) adopting a deep-sequencing approach (about 150,000 fragments/samples).

Paired end reads were trimmed at the first base with a quality score under 20, to keep only high-accuracy sequences. Afterwards, trimmed reads were merged with their counterparts, and the resulting sequences were used as input for metagenomic analysis on the ITS region, an average merging rate of 92% was obtained. As the number of pre-treated fragments per sample varied greatly, samples with more than 100,000 fragments were randomly subsampled to this amount, consequently for each sample there were from 68,626 to 100,000 fragments.

The analysis was carried out using QIIME2 software (QIIME2.2023-9) (Bolyen et al., 2019). Sequences trimmed for ITS primers Cutadapt ranged from 65,248 to 98,551. Afterwards such fragments were checked in terms of quality and all of them presented quality score higher than 20. Reads passing the denoising and chimera detection with DADA2 pipeline to the classification process ranged from 63,419 to 98,340; afterward 4,567 Amplicon Sequence Variants (ASVs) were identified with a minimum and maximum length of 66 and 458 nucleotides, respectively and a mean length of 323 nucleotides (**Table 2**). Raw forward and reverse reads were deposited in Genbank SRA database under the PRJNA1097375 Bioproject accession number.

**Table 2:** Overview of sequencing and data processing preliminary steps.

Sequence parameter	Value
Assembled reads	∼7.2 million
Rarified reads	∼4.34 million
ASVs	4567 (4501 after rarefaction)
ASVs annotated to:	
Species	2182 (47.7%)
Genus	2250 (49.26%)
Family	2797 (61.24%)
Order	3519 (77.05%)
Class	3790 (82.98%)
Phylum	4116 (90.1%)

ITS2 sequences were trimmed for 5.8S and SSU flaking fragments using ITSx (Bengtsson-Palme et al., 2013) implemented in the Galaxy platform (The Galaxy Community, 2024). Processed ASVs were then classified using the Naïve-Bayes classifier trained on Unite-v9-99_ITS database. Taxonomic assignment p-confidence threshold was set to 0.8 increasing the value of 0.7 used as default in the classifier. A total of 568 genera and 793 species were identified by sorting the ITS data (**Table 2**).

Subsequent analyses were performed in the R environment using QIIME2 exported files, using a set of functions implemented in several packages including Phyloseq (McMurdie and Holmes, 2013), microbiomeutilities (Shetty et al., 2018), MicrobiomeMarker (Cao et al., 2022), Vegan (Oksanen et al., 2024), DeSeq2 (Love et al., 2014), and Metacoder (Foster et al., 2017). Rarefaction value was determined using accumulation curves (**Supplementary Figure 1**) and set to 0.9X of the sample with lowest reads count (63,419), so building a final dataset of 57,077 merged reads per sample, the dataset contained 4501 ASV.

Beta-diversity was estimated using Bray–Curtis dissimilarity matrices calculated from normalized abundance tables. Differences in community composition among experimental groups were assessed by permutational multivariate analysis of variance (PERMANOVA) using the adonis2 function implemented in the vegan package. Both univariate and multivariate models were tested, including additive and interaction terms where appropriate. Variables tested included cluster, health status, and organ type.

PERMANOVA analyses were performed with 999 permutations using marginal tests (by = “margin”), allowing the effect of each explanatory variable to be evaluated while controlling for the others included in the model. For each term, F statistics, R² values, and permutation-based p-values were extracted.

To account for potential non-independence among samples, the workflow included an optional stratified permutation framework. Nesting was assessed by determining whether levels of a variable occurred exclusively within a single level of a candidate blocking factor (e.g., tree). Variables fully nested within the selected stratum were flagged because their effects cannot be independently tested under constrained permutations.

To control for multiple testing, *p*-values obtained across all PERMANOVA terms were adjusted using the false discovery rate (FDR) correction method. Terms with adjusted *p*-values < 0.05 were considered statistically significant.

For significant univariate PERMANOVA results, homogeneity of multivariate dispersion was evaluated using the betadisper and permutest functions from the vegan package. Dispersion tests were conducted with 999 permutations. Significant dispersion heterogeneity was interpreted as a potential violation of PERMANOVA assumptions, and corresponding results were therefore interpreted with caution.

To assess the robustness of PERMANOVA results to individual samples, a leave-one-out analysis was conducted at the tree level. Each tree was iteratively removed from the dataset, the Bray–Curtis distance matrix recalculated, and PERMANOVA repeated on the reduced dataset. Changes in p-values and model significance following removal of individual trees were recorded to identify potentially influential samples.

FUNGuild (Nguyen et al., 2016) was employed to predict the functional profiles of fungal community, after dataset rarefaction (4501 total ASVs). Only “highly probable” or “probable” annotations were considered, for a total of 2021 ASVs. Fungi were categorized into different trophic modes and guilds according to their nutritional mode across various clusters and tree health status. FungalTraits (Põlme et al., 2020) was also used to provide further putative functional annotations.

## 3 Results

### 3.1 Overview of *Abies nebrodensis* mycobiota

In this study, the fungal community associated with the relic population of *A. nebrodensis* was analyzed in a single spring sampling campaign, using a metabarcoding approach based on the ITS2 region.

A total of 4501 ASVs were obtained from the DNA of the 76 samples examined; most of them (77.9%) belonged to the phylum Ascomycota, followed by Basidiomycota (11.7%), Chytridiomycota (0.44%), Olpidiomycota (0.07%), Mucoromycota (0.02%), and unclassified fungi (9.9%) (**Supplementary Figure 2**). The complete mycobiome was composed by 26 classes of fungi, belonging to 77 orders, 184 families, 317 genera, and 285 species.

### 3.2 Comparative diversity of phyllospheric and endospheric fungal communities associated with *A. nebrodensis*

The comparison between surface sterilized (SS) and non-sterilized (NS) samples may offer a view of the endospheric communities compared to those of the entire phyllosphere. We preferred to use the term ‘endospheric’, also adopted by other authors (Qian et al., 2019: Blaszczyk et al., 2021), which refers to fungi that colonize internal tissues, rather than ‘endophytic’, which refers to fungi that colonize living plant tissues without causing apparent symptoms, according to Wilson (1995).

**Figure 3:**
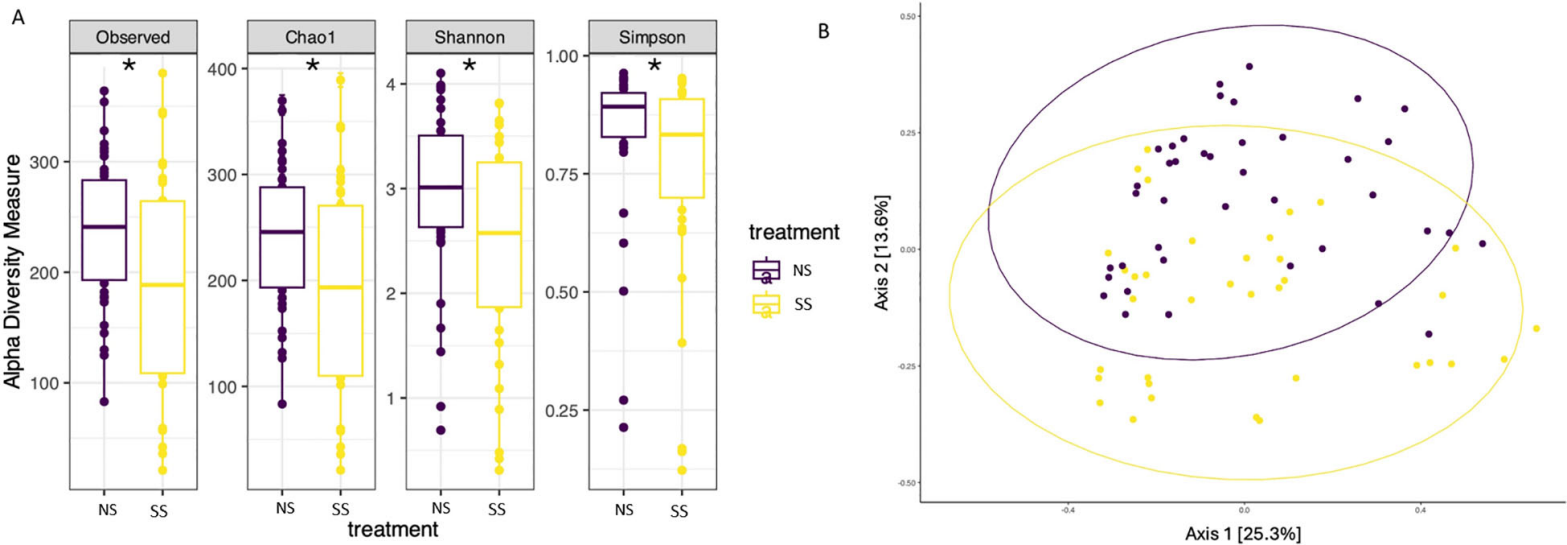
**(A)** Alpha diversity indices of the total fungal communities of Non-Sterilized (NS) and Sterilized (SS) samples (* indicates *p* < 0.05); **(B)** Bray-Curtis non phylogenetic beta-diversity between the entire phyllosphere and endosphere communities.

Permanova analysis clearly suggested that the surface sterilization treatment significantly affects the fungal communities (*p* < 0.01, see **Supplementary Table 1**) and different Alpha-diversity indices were obtained from NS and SS samples, as shown in **Figure 3A**. All the indices consistently suggested a significant (*p* < 0.05) higher diversity of the full phyllospheric fungal community compared to the endospheric community.

Non phylogenetic Bray-Curtis β-diversity between NS and SS showed (**Figure 3B**) that the two communities have statistically different compositions, though partially overlapping (Permanova with 999 permutations *p* = 0.001, betadisper *p* = 0.927).

Interestingly, the endospheric fungal community was not merely a subset of the full phyllospheric community, but it contained unique ASVs. Notably, 936 ASVs were shared by NS and SS, while 1806 and 1759 were unique to the SS and NS communities, respectively (see also **Supplementary Figure 3**).

#### 3.2.1 Diversity of the *A. nebrodensis* full phyllospheric fungal community

**Figure 4:**
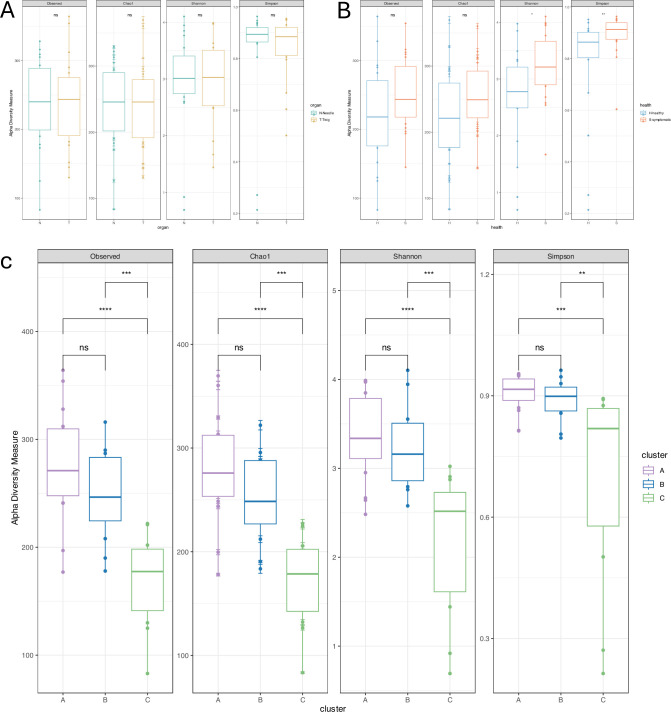
Alpha diversity indices of the entire phyllosphere fungal communities: **(A)** organ specific differences (needle/twig); **(B)** health status (healthy/symptomatic); **(C)** cluster of plants (A/B/C). *p < 0.05, **p < 0.01, ***p < 0.001, ****p < 0.0001.

Alpha-diversity (**Figure 4A**) resulted not significantly different when the two organs (N = Needle vs. T = Twigs) were compared among NS samples. As regards comparison between healthy (H) and symptomatic (S) samples, the observed number of ASVs and Chao1 indexes were not significant; conversely, Shannon and Simpsons indexes resulted significantly higher in S samples (**Figure 4B**). Overall, the phyllospheric fungal community of S samples showed a higher alpha-diversity. When comparing the three groups of trees, group C, which included trees living isolated in the harshest ecological conditions, showed a significantly lower alpha-diversity (**Figure 4C**).

**Figure 5:**
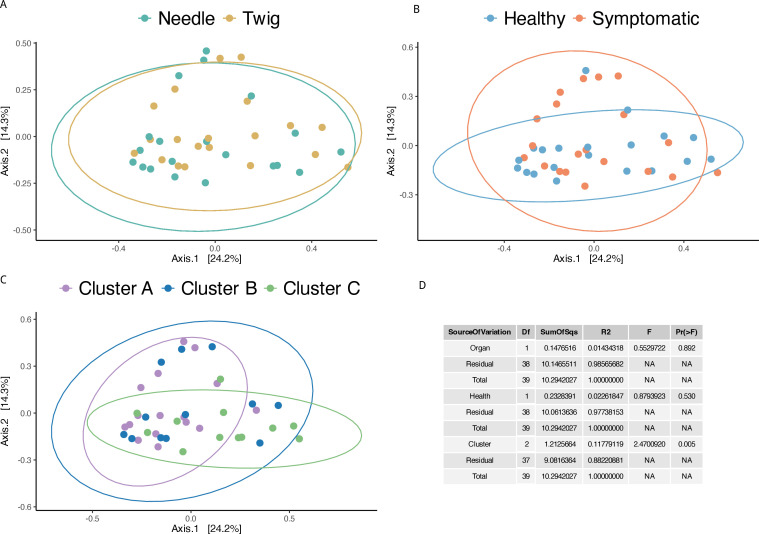
Bray-Curtis non-phylogenetic beta-diversity of the entire phyllosphere fungal communities: **(A)** tissue specific differences (needle/twig); **(B)** health status (healthy/symptomatic); **(C)** cluster of trees (A/B/C); **(D)** Permanova with 999 permutations.

The analysis of Bray–Curtis beta-diversity (**Figure 5**) suggested that, among the analyzed factors, only habitat (tree cluster) significantly affected the fungal communities of the Sicilian fir phyllosphere (PERMANOVA *p* = 0.008). Nevertheless, the variation in dispersion among clusters was not statistically significant (betadisper *p* = 0.112), suggesting that the observed differences were primarily associated with community composition rather than unequal within-group dispersion. Leave-one-out analyses demonstrated the robustness of the global PERMANOVA result, as significance was retained after removal of each individual tree, supporting the stability and consistency of the observed cluster effect.

#### 3.2.2 Diversity of the *A. nebrodensis* endospheric fungal community

Alpha-diversity resulted not significantly different among leaves and twigs, healthy and symptomatic samples and groups of trees. This result is also consistent with the beta-diversity analysis (**Supplementary Figures 4**, **5**).

### 3.3 Taxonomic composition of *A. nebrodensis* fungal community

As no statistically significant differences were observed between the tissue types (twigs and needles), the variable “organ” was excluded from subsequent analyses, focusing on assessment of the fungal community taxonomy in relation to the remaining variables.

**Figure 6:**
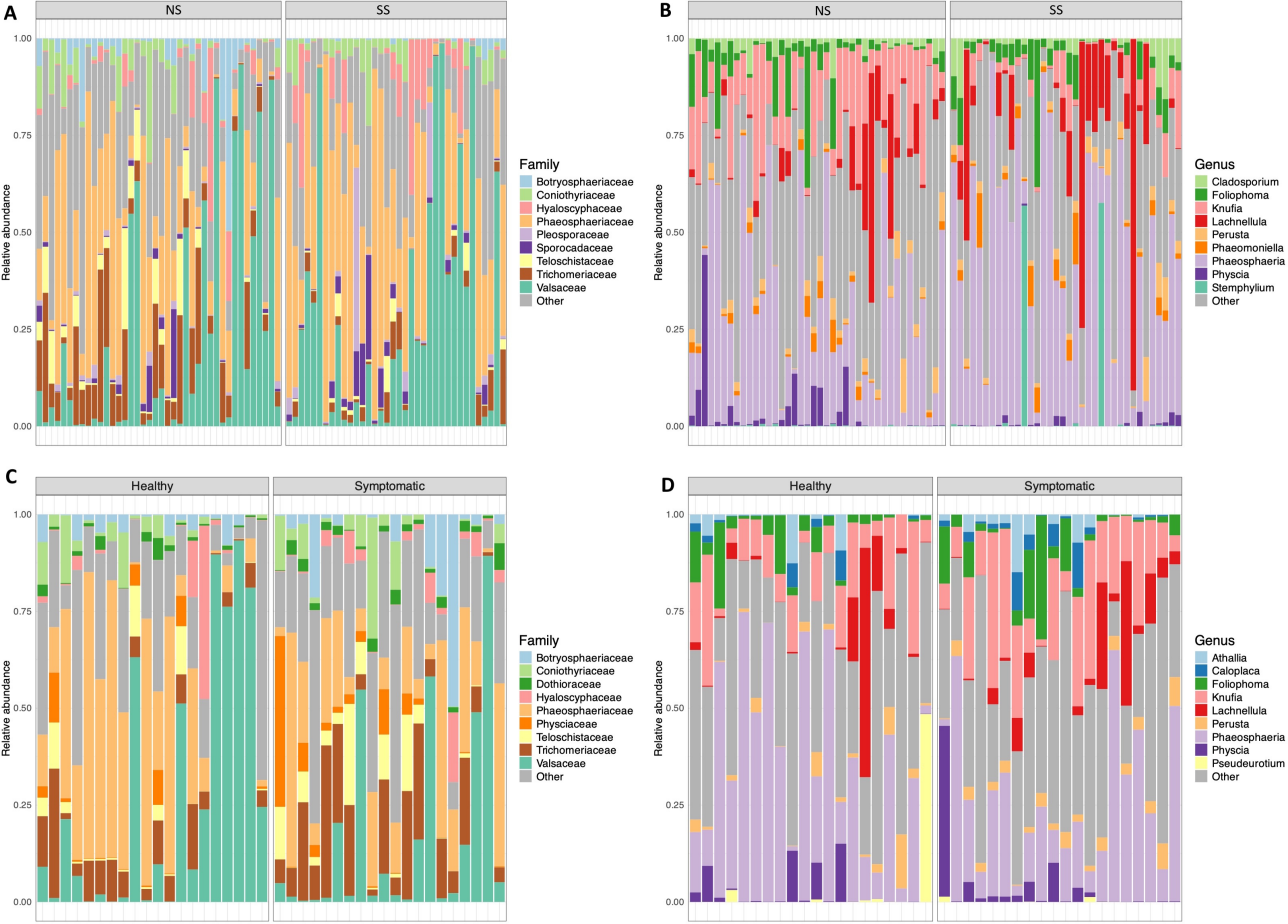
Upper panel: taxa barplot depicting the composition (relative abundance) of the fungal phyllospheric (NS) and endospheric (SS) communities at family **(A)** or genus **(B)** levels. Lower panel: taxa barplot depicting the composition of the phyllosphere fungal communities (NS) associated to heathy or symptomatic samples, at family **(C)** or genus **(D)** levels. Only the 9 most abundant families or genera are reported. A category “other” sums all remaining families (or genera).

The barplots in **Figure 6** display the ASVs taxonomically resolved at family (**Figures 6A, C**) and genus levels (**Figures 6B, D**) respectively divided between NS/SS samples or healthy/symptomatic samples. For the family/genus-level barplots, only the top nine families or genera ranked by mean relative abundance are shown individually, while all remaining families are grouped into the category “Other”. The most abundant families in the total phyllosphere fungal community were: Valsaceae (mean relative abundance 17%), Phaeosphaeriaceae (12%), Trichomeriaceae (6%), Botryosphaeriaceae (5%), and Teloschistaceae (4%). The complete table of relative abundances is available in Supplementary file 1. Among the most abundant families, Trichomeriaceae (*p* < 0.01), Botryosphaeriaceae (*p* < 0.01), Teloschistaceae (*p* < 0.05), and Lecanoraceae (*p* < 0.05) were statistically more common in fungal communities of the full phyllosphere, while Phaeosphaeriaceae (*p* < 0.05), Sporocadaceae (*p* < 0.05), and Godrionaceae (*p* < 0.05) were more abundant in endospheric communities (**Supplementary Figure 6**). It should be noted that some abundant ASVs were not taxonomically classified at the family or genus level and are therefore not displayed in the figure. Nonetheless, these unclassified ASVs were commonly detected in both total and endospheric communities, showing different relative abundances between the NS and SS groups, in some cases with statistical significance (**Supplementary Figure 7**).

The comparison between healthy and symptomatic samples showed that abundance of the most frequent families in the full phyllospheric community (Valsaceae, Phaeosphaeriaceae, and Trichomeriaceae) was not significantly different (**Figure 6C**; **Supplementary File 2**, **Supplementary Figure 8**), except for Dothioraceae which resulted significantly more frequent in symptomatic samples (p < 0.01) (**Supplementary Figure 8**) though a high degree of variability in community composition resulted among samples. At the genus level (**Figure 6D**), Phaeosphaeria (14%), Knufia (4.5%), Lachnellula (2.6%), and Foliophoma (1.8%) were among the most prevalent taxa in healthy samples. Phaeosphaeria (9.7%) and Knufia (8%) were also dominant in symptomatic samples along with Foliophoma (2.6%) and Physcia (2.3%). Only the genus Perusta resulted significantly (p < 0.01) more abundant in symptomatic samples (**Supplementary Figure 9**). The lichen forming fungi Athallia (p < 0.05), Lecidella (p < 0.01), Caloplaca (p < 0.05), and the genus Knufia (p < 0.01) were significantly more represented in the full phyllosphere community (see also **Supplementary Figure 10**).

**Figure 7:**
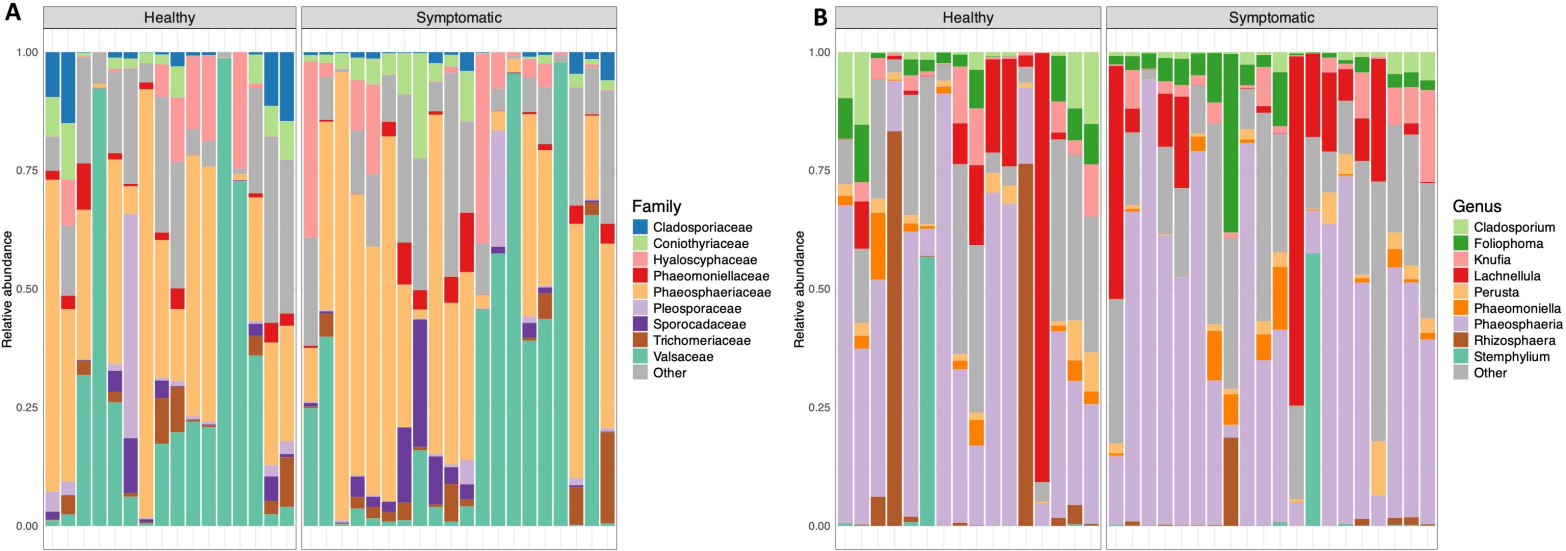
Taxa bar plot depicting the composition (relative abundance) of the endospheric (SS) communities of healthy and symptomatic samples. Only the 9 most abundant families **(A)** or genera **(B)** are reported, the category “Other” includes all remaining families (or genera).

As regards the endospheric community, Valsaceae, Phaeosphaeriaceae, and Hyaloscyphaceae are the most common taxonomically resolved families, which together account on average for more than half of the total fungal communities in both healthy and symptomatic samples (**Figure 7A**; **Supplementary Figure 11**). At genus level, Phaeosphaeria and Lachnellula were the most abundant in both groups (**Figure 7B**; **Supplementary File 3**) along with an unresolved group of ASVs belonging to Valsaceae. No statistically significant differences were observed between healthy and symptomatic samples neither at family nor at genus level (**Supplementary Figures 11**, **12**).

Focusing on symptomatic samples, Phaeosphaeriaceae resulted significantly (*p* < 0.05) more abundant in S samples than in the NS samples along with Sporocadaceae (*p* < 0.05). Conversely, Botryosphaeriaceae, (*p* < 0.01), Trichomeriaceae (*p* < 0.01), Lecanoraceae (*p* < 0.05), and Telochistaceae (*p* < 0.01), resulted more frequent in the NS samples (**Supplementary Figure 13a-b**, **Supplementary File 4**).

### 3.4 Variability of *A. nebrodensis* mycobiota in relation to habitat conditions

**Figure 8:**
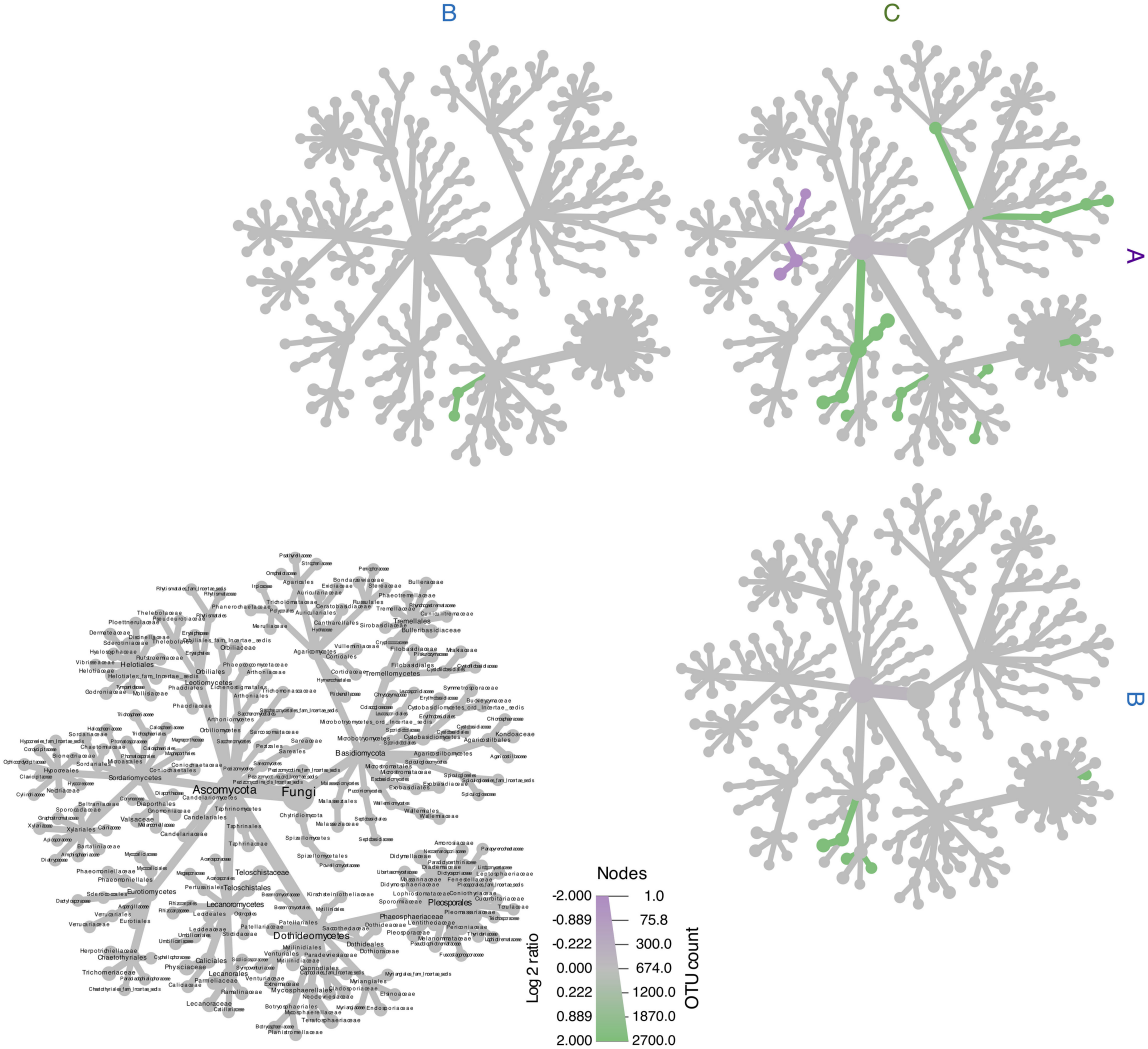
Differential heat-trees comparing the full phyllosphere communities of trees from the clusters A, B, and C. Only the families that are differentially abundant (*p* < 0.01) are colored. The heath-tree shows differential abundance across the three pairwise comparisons (A vs B, B vs C, and A vs C). For each comparison, positive log-fold change values (green) indicate taxa more abundant in the first group listed, whereas negative values (purple) indicate taxa more abundant in the second group. Consequently, the reference group changes across comparisons (A in A vs B and A vs C; B in B vs C).

When comparing the three groups of trees, **Figure 8** displays a differential abundance heat-tree (*p* < 0.01) of the total fungal phyllosphere community, highlighting how the mycobiome of trees in cluster C differed significantly from that in cluster B, and especially from those in cluster A. Valsaceae are highly more represented in cluster C compared to cluster A, along with the less abundant family Calosphaeriaceae. In contrast, the families Dothideaceae, Kondoaceae, Myriangiaceae, Parmeliaceae, Physciaceae, Teloschistaceae, and Venturiaceae are more abundant in cluster A than in cluster C. Venturiaceae is the only differentially represented family (*p* < 0.01) among cluster A and B. At the genus level, *Athallia*, *Caloplaca*, *Lecidella*, *Phaeomoniella*, *Physcia*, *Rhizosphaera*, and *Xanthoria* were significantly more abundant in groups A and B compared to C (*p* < 0.05 and *p* < 0.01, see **Supplementary Figure 14**, **Supplementary File 5**). This trend was also observed for additional ASVs that could not be resolved at the genus level.

In the endospheric communities, Phaeosphaeriaceae (27%, 28%, and 13%, respectively) and Valsaceae (14%, 24%, and 37%, respectively) are the most abundant families in all the three groups of trees (**Supplementary Figure 15**, **Supplementary File 6** for the complete table of frequencies). No significant differences in abundance were found for any taxonomic group (orders, families, genera) in the endospheric communities among the three groups of trees.

### 3.5 *Abies nebrodensis* core mycobiota

*A. nebrodensis* core microbiota was identified at the ASV level using an abundance–occurrence approach (Neu et al., 2021), with a minimum relative abundance threshold of 1% to include low-abundance fungi and occurrence in at least 50% of the samples.

The core mycobiota (**Table 3**) of the full phyllospheric community is composed by 34 ASVs, belonging to the families Botryosphaeriaceae, Cladosporiaceae, Trichomeriaceae, Valsaceae, Dothideaceae, Dothioraceae, Hyaloscyphaceae, Phaemonelliaceae, Coniothyriaceae, Phaeosphaeriaceae, Teloschistaceae, Chaetothyriales_Incertae_sedis. The occurrence of the core microbiota, defined as the cumulative presence of the ASVs constituting the core, spanned from a minimum of 39% to a maximum of 96% across all samples, with an average occurrence of 78% ± 13% (**Supplementary File 7**).

**Table 3:** Composition of *A. nebrodensis* phyllosphere core fungal microbiota.

ASV	Phylum	Class	Order	Family	Genus
NS10	Ascomycota	Dothideomycetes	Botryosphaeriales	Botryosphaeriaceae	
SH14	Ascomycota	Dothideomycetes	Capnodiales	Cladosporiaceae	*Cladosporium*
SH15	Ascomycota	Dothideomycetes	Capnodiales	Cladosporiaceae	*Cladosporium*
NS1	Ascomycota	Eurotiomycetes	Chaetothyriales	Trichomeriaceae	*Knufia*
SH3	Ascomycota	Eurotiomycetes	Chaetothyriales	Trichomeriaceae	*Knufia*
SH13	Ascomycota	Eurotiomycetes	Chaetothyriales	Chaetothyriales_fam_Incertae_sedis	*Sarcinomyces*
SH1	Ascomycota	Sordariomycetes	Diaporthales	Valsaceae	
SH2	Ascomycota	Sordariomycetes	Diaporthales	Valsaceae	
NS5	Ascomycota	Dothideomycetes	Dothideales	Dothideaceae	*Rhizosphaera*
SH11	Ascomycota	Dothideomycetes	Dothideales	Dothioraceae	*Perusta*
SH12	Ascomycota	Dothideomycetes	Dothideales	Dothioraceae	*Perusta*
SH16	Ascomycota	Leotiomycetes	Helotiales	Hyaloscyphaceae	*Lachnellula*
SH4	Ascomycota	Eurotiomycetes	Phaeomoniellales	Phaeomoniellaceae	*Phaeomoniella*
NS4	Ascomycota	Dothideomycetes	Pleosporales	Coniothyriaceae	*Foliophoma*
SH7	Ascomycota	Dothideomycetes	Pleosporales	Coniothyriaceae	*Foliophoma*
SH9	Ascomycota	Dothideomycetes	Pleosporales	Phaeosphaeriaceae	*Phaeosphaeria*
NS7	Ascomycota	Lecanoromycetes	Teloschistales	Teloschistaceae	*Athallia*

Out of the total 34 ASVs identified, only the 17 ASVs annotated at least at family level are reported. Original ASV identifiers were replaced with shorter labels to enhance figure and table readability: ASV starting with NS are exclusive of the full phyllosphere core, ASV starting with SH are shared among the full phyllosphere and endospheric core (see **Supplementary Table 5**).

**Figure 9:**
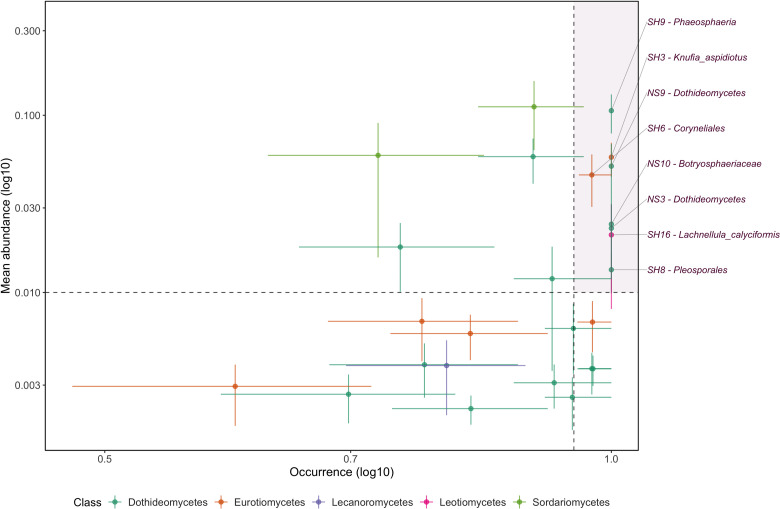
Abundance-occurrence plot of *A. nebrodensis* phyllosphere core microbiota. Taxonomic assignment is reported only for AVS with at least 90% occurrence and 1% abundance; only ASVs assigned at least at Class level are included for graphical clarity (see Supplementary file 7). Core microbiome ASV names were recoded to shorter identifiers to improve clarity.

An abundance-prevalence plot for the phyllosphere core microbiota is reported in **Figure 9**, showing the taxonomic affiliation of the most abundant and commonly found (>90% of samples) ASVs building the core mycobiota. An ASV belonging to the genus *Phaeosphaeria* is the most represented, accounting for 10% of the total microbiota in the 100% of the samples. Other ASVs showed 100% occurrence were assigned to the genera *Knufia* and *Lachnellula*, the family Botryosphaeriaceae, the class Dothideomycetes, and the order Pleosporales.

Various melanized fungi are also represented: *Knufia*, *Perusta*, *Cladosporium*, *Alternaria*, *Sarcinomyces*, along with *Lachnellula*, *Foliophoma*, *Phaemoniella* and *Athallia* (**Table 3**).

The endospheric core mycobiota (SS samples) consists of a smaller set of ASVs (22) and exhibits lower diversity compared to the core mycobiota of the full phyllosphere (**Table 4**). Its occurrence ranged from 29% to 98% across all samples, with an average occurrence of 78% ± 15% (supplementary file 7). The core mycobiota communities of NS and SS samples share 17 ASVs corresponding to 50% of the phyllospheric core and 77% of the endospheric core, 17 are exclusive of NS and 5 of the SS mycobiota, respectively.

**Table 4:** Composition of *A. nebrodensis* endospheric core fungal microbiota.

ASV	Phylum	Class	Order	Family	Genus
SH14	Ascomycota	Dothideomycetes	Capnodiales	Cladosporiaceae	*Cladosporium*
SH15	Ascomycota	Dothideomycetes	Capnodiales	Cladosporiaceae	*Cladosporium*
SH3	Ascomycota	Eurotiomycetes	Chaetothyriales	Trichomeriaceae	*Knufia*
SH13	Ascomycota	Eurotiomycetes	Chaetothyriales	Chaetothyriales_fam_Incertae_sedis	*Sarcinomyces*
SH1	Ascomycota	Sordariomycetes	Diaporthales	Valsaceae	
SH2	Ascomycota	Sordariomycetes	Diaporthales	Valsaceae	
SS5	Ascomycota	Dothideomycetes	Dothideales	Dothioraceae	*Perusta*
SH11	Ascomycota	Dothideomycetes	Dothideales	Dothioraceae	*Perusta*
SH12	Ascomycota	Dothideomycetes	Dothideales	Dothioraceae	*Perusta*
SH16	Ascomycota	Leotiomycetes	Helotiales	Hyaloscyphaceae	*Lachnellula*
SH4	Ascomycota	Eurotiomycetes	Phaeomoniellales	Phaeomoniellaceae	*Phaeomoniella*
SH7	Ascomycota	Dothideomycetes	Pleosporales	Coniothyriaceae	*Foliophoma*
SS2	Ascomycota	Dothideomycetes	Pleosporales	Pleosporaceae	*Alternaria*
SS3	Ascomycota	Dothideomycetes	Pleosporales	Phaeosphaeriaceae	*Phaeosphaeria*
SS4	Ascomycota	Dothideomycetes	Pleosporales	Phaeosphaeriaceae	*Phaeosphaeria*
SH9	Ascomycota	Dothideomycetes	Pleosporales	Phaeosphaeriaceae	*Phaeosphaeria*
SS1	Ascomycota	Sordariomycetes	Xylariales	Sporocadaceae	

Out of the total 22 ASVs identified, only the 17 ASVs annotated at least at family level are reported. Original ASV identifiers were replaced with shorter labels to enhance figure and table readability: ASV starting with SS are exclusive of the endospheric core, ASV starting with SH are shared among the full phyllosphere and endospheric core (see **Supplementary Table 5**).

**Figure 10:**
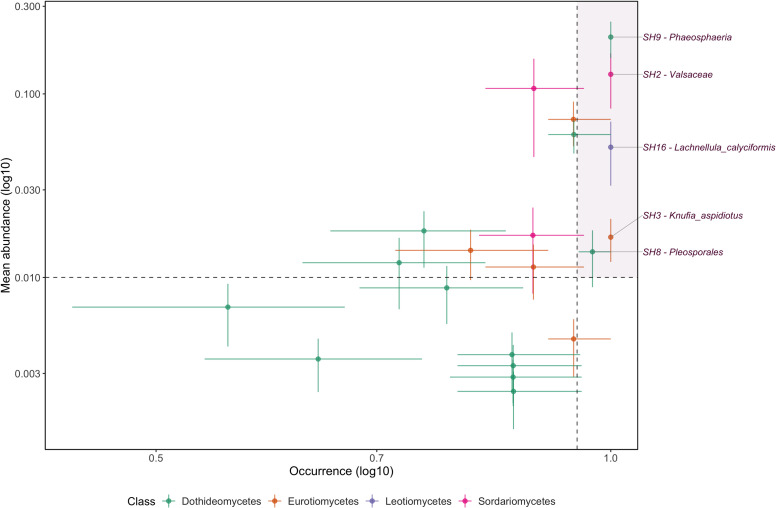
Abundance-occurrence plot of *A. nebrodensis* endospheric core microbiota. Taxonomic assignment is reported only for AVS with at least 90% occurrence and 1% abundance; only ASVs assigned at least at Class level are included for graphical clarity (see Supplementary file 8 for the full data table). Core microbiome ASV names were recoded to shorter identifiers to improve clarity.

The fungal families represented in the endospheric core microbiota were the same of the full phyllospheric mycobiota, except for Botryosphaeriaceae and Teloschistaceae (genus Athallia) which were depleted in the endospheric core (**Table 4**, **Figure 10**). An ASV referring to Sporocadaceae was not represented in the full phyllosphere.

The genus Phaeosphaeria, already dominant in the full phyllospheric core, is even more abundant (20% relative abundance) and widely present (100% occurrence) in the endospheric communities.

The detailed composition of the endospheric core mycobiota in healthy and symptomatic samples is reported in **Supplementary Tables 2**, **3**, respectively. In healthy samples the core mycobiota consists of 22 ASVs of three classes (Dothideomycetes, Eurotiomycetes, Sordariomycetes). At the genus level, two ASVs are affiliated with Cladosporium, three with Perusta, and three with Phaeosphaeria. The core microbiota represents as average the 73% (± 18%) of the total community with a maximum of 98% and a minimum of 30% (Supplementary file 8). For the symptomatic samples the core is composed by 24 ASVs belonging to the same taxa reported in healthy samples, except for one ASV affiliated with the genus Lachnellula (family Hyaloscyphaceae), that is unique to the core of the symptomatic samples, while an ASV affiliated with Didymellaceae is unique of healthy samples. Among the two cores of healthy and symptomatic samples, 19 ASVs (86% and 79% of the respective total cores) are shared while two are exclusive of the healthy samples core. These unique ASVs are highlighted in bold in **Supplementary Tables 2**, **3**. In symptomatic samples, the core microbiota represents as average the 81% (± 11%) of the total communities with a maximum of 98% and a minimum of 64% (Supplementary file 8).

### 3.6 Functional prediction of fungal community

**Figure 11:**
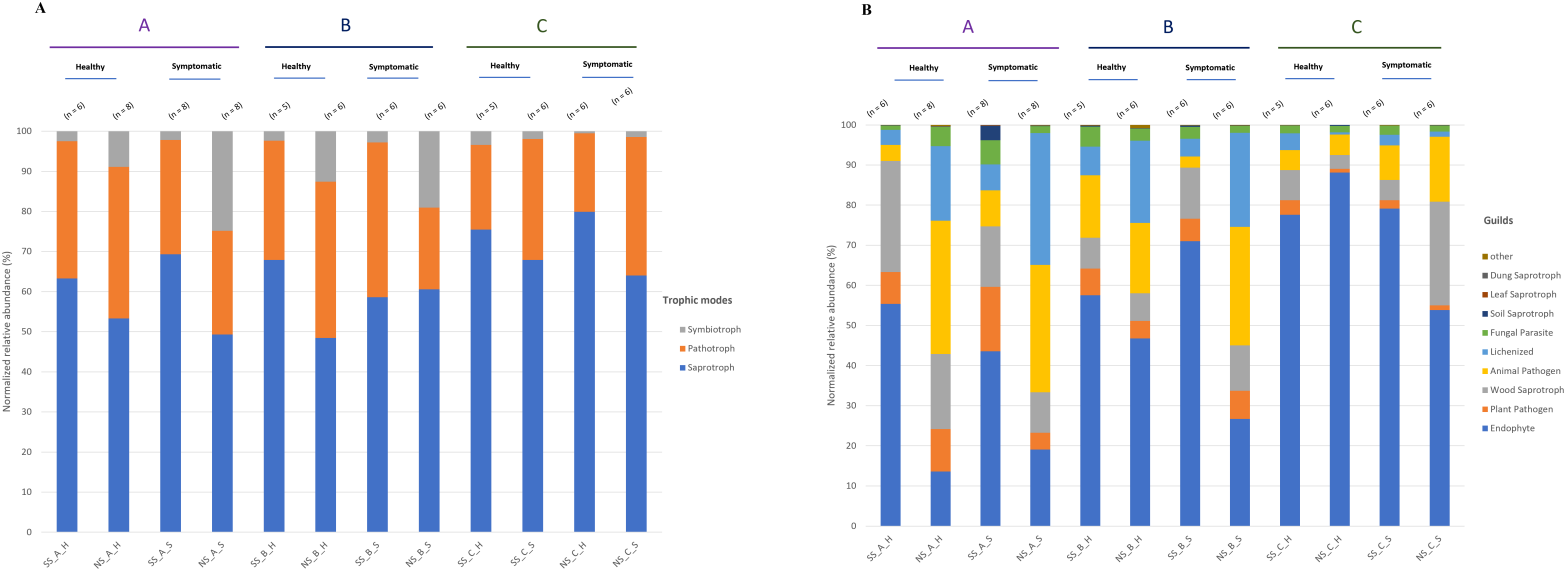
Putative functional guild and trophic mode assignments of ASVs from the *Abies nebrodensis* phyllosphere, inferred from taxonomic identity using FUNGuild. **(A)** Relative abundance of trophic modes; **(B)** Relative abundance of guilds. Only the top 9 guilds are shown. A, Cluster A; B, Cluster B; C, Cluster C; SS, Surface-Sterilized; NS, Non-Surface-Sterilized; H, Healthy; S, Symptomatic.

To have an initial cue of the potential lifestyle of fungal species, the putative ecological and functional role of the ASVs obtained from the sampled trees was estimated using the FUNGuild annotation and cross-checked with the curated FungalTraits database. Based on these annotations, fungal communities were provisionally categorized into trophic modes and functional guilds (FUNGuild, **Figure 11A, B**) and into primary lifestyles (FungalTraits, **Supplementary Figures 16A, B**). As shown in **Figure 11A**, most ASVs were classified as taxa frequently associated with saprotrophic strategies, accounting for approximately 48–80% of the total assigned fungal community among the various samples, followed by ASVs classified as taxa with pathotrophic lifestyles. Taxa classified as fungi presenting symbiotrophic lifestyle, represented a comparatively minor fraction of the community, contributing less than 4% in most sterilized samples.

Within the functional guilds (**Figure 11B**), endophyte-assigned taxa were the most frequently assigned guilds across the samples. Their abundance was particularly high in Cluster C, where they exceeded 77% in healthy (H) samples and reached 88.11% in the full phyllosphere community of healthy samples in group C (NS_C_H). Taxa assigned to the wood saprotroph guild were the second most abundant category, especially in trees of the group A, though they are ubiquitously spread among all samples. The FungalTraits analysis confirmed this trend: across all clusters, saprotrophic lifestyles (including litter, soil, wood, and unspecified saprotrophs) represented a substantial proportion of the annotated community. Interestingly, plant pathogen guild was present in all clusters but remained subdominant.

## 4 Discussion

The plant microbiota comprises diverse, taxonomically structured microbial communities colonizing all outer and inner tissues. This study provides the first insight, based on single sampling campaign, into the phyllosphere fungal communities of *A. nebrodensis* and the changes they undergo based on organ (needle/twig), health status of tissues (healthy/symptomatic) and habitat conditions. Our approach was fundamentally pathological, focusing on visible disorders in the crowns, partly to expand upon the findings of the previous study based on phytosanitary inspections and fungal isolations (Frascella et al., 2024). High-throughput sequencing yielded 4,501 ASVs, demonstrating good sequencing sensitivity and enabling detection of rare taxa. Among Ascomycetes (approximately 78% of all ASVs), the most represented classes were Dothideomycetes (41%), Sordariomycetes (22%), Eurotiomycetes (13%), and Leotiomycetes (4%). Basidiomycetes (11.7% of ASVs) showed low abundance (around 1%). Rare ASVs representing Chytridiomycota, Olpidiomycota, and Mucoromycota were also detected; these taxa are known for their low occurrence in forest trees (Schon et al., 2023).

### 4.1 Full phyllospheric and endospheric community

Metabarcoding of endospheric fungi was achieved via surface sterilization (SS) of plant samples prior to DNA extraction. Comparison between SS and non-sterilized (NS) samples offers insights into the selection of endospheric mycobiota (Kohler et al., 2025). The 936 ASVs shared between NS and SS samples accounted for 98% of total rarefied reads. ASVs exclusive to the epiphytic community (1,759) were derived by subtracting SS-sample ASVs from the full NS phyllosphere. Both unique full phyllospheric and endospheric communities had very low frequency (1.05% and 0.88%, respectively). Unlike findings by Kohler et al. (2025) and Yao et al. (2019), ASV richness did not decrease in SS samples; surface sterilization affected relative abundance but not richness.

Both α- and β-diversity were significantly higher in the full phyllosphere than in the endospheric community. However, the endospheric community was not a simple subset of the full phyllospheric community: 1,806 ASVs were exclusive to the endospheric assemblage. Their very low frequency suggests they became detectable only after surface sterilization removed most epiphytic fungi and may represent at least partially a technical driven result rather than a biological signal, considering that often a species-specific procedure for surface-sterilization is required to guarantee complete removal of surface organism (and the DNA associated) (Sahu et al., 2022). This potential bias underlines the importance of adequate sequencing depth in complex biomes.

The greater biodiversity of the full phyllosphere compared to the endosphere likely reflects the higher species richness typical of epiphytic communities, which are more exposed to aerial inoculum and stochastic events (Chen et al., 2025; Köhler et al., 2025; Gomes et al., 2018). Endophytic fungi, by contrast, are highly specialized within the more sheltered internal plant environment, subject to deterministic plant–fungus interaction dynamics, including host selection and immune evasion (Li et al., 2025b; Brodde et al., 2025; Trivedi et al., 2022). The dominant families and genera were largely the same across NS and SS samples, with minor frequency shifts. Valsaceae and Phaeosphaeriaceae dominated the full phyllospheric community, with the latter showing higher abundance in SS samples along with Sporocadaceae. *Phaeosphaeria* was the most represented genus, showing twice the frequency in SS compared to NS samples (22% vs. 11%). Some genera, i.e. *Knufia* and fungi that form lichens, like *Athallia*, *Lecidella*, and *Caloplaca* showed significantly higher frequency in NS samples than in S samples.

### 4.2 Organ effect

No significant differences emerged in either α- or β-diversity between needles and twigs, in either the full phyllospheric or endospheric community. While fungal richness and diversity can vary between plant compartments (Cregger et al., 2018), usually comparisons among organs involve different niches (root/stem/leaves) rather than fine scale variations. Nevertheless, some studies report higher richness in twigs and others in leaves, depending on the specific plant species and environment (Gomes et al., 2018; Costa et al., 2021; Daghino et al., 2022).

### 4.3 Health status effect

Comparison between healthy (H) and symptomatic (S) samples revealed no significant differences in ASV richness in the full phyllospheric community, but significantly higher diversity in symptomatic samples based on Shannon and Simpson indices. No significant diversity differences were instead found for the endospheric community between H and S samples. Among families, only Dothioraceae showed significantly higher frequency in S samples, while Valsaceae, Phaeosphaeriaceae, and Trichomeriaceae showed no abundance variation. Among the most abundant genera, *Phaeosphaeria*, *Knufia*, *Lachnellula*, and *Foliophoma* dominated both sample types, with only *Perusta* showing significantly higher abundance in symptomatic samples.

Despite the general absence of significant differences, DESeq2 differential analysis of endospheric communities showed enrichment in symptomatic samples of *Phaeosphaeria*, *Allelochaeta*, *Herpotrichia*, *Caloplaca*, *Orbilia*, *Didymella*, *Tumularia*, and *Jattaea*, as well as Valsaceae and Phaeosphaeriaceae members. The core mycobiome analysis confirmed that dominant taxa showed no significant abundance differences between H and S samples.

HTS metabarcoding did not reveal primary pathogens, consistent with previous studies and confirming environmental stress as the main cause of shoot and needle blight in *A. nebrodensis* (Frascella et al., 2024). As reported in other systems, the presence of a disease may increase diversity and richness of the fungal community (Liu et al., 2022; Menkis et al., 2015; Li et al., 2025). The greater fungal diversity in symptomatic samples may reflect loss of resistance factors in damaged tissues, creating new niches (De Mandal and Jeon, 2023), or may be related to altered microbial community stability following disease (Brodde et al., 2025).

### 4.4 Habitat effect

The main effect on both α- and β-diversity and composition of the phyllosphere fungal community of *A. nebrodensis* is due to variations of habitat ecological factors. Trees were divided into three groups: Group A (“mixed rocky slope”: rocky slopes, shallow nutrient-poor soils, strong sunlight and wind, broadleaves nearby); Group B (“closed-canopy beech forest”: trees within beech grove, protected from wind, higher humidity, richer soils, diverse understory); Group C (“exposed ridge”: isolated trees near ridges, rocky shallow soils, maximum exposure to wind, storms, hail and sunlight, no neighboring trees). Both α-and β-diversity in the full phyllosphere were significantly reduced in Group C. This effect did not occur in the endospheric community, which showed no diversity differences among the three groups.

Several studies have shown that diversity and community composition of phyllosphere epiphytic and endophytic fungi are diverse due to the different microenvironments in which they live (Santamaría and Bayman, 2005; Coleman-Derr et al., 2016; Gomes et al., 2018; Osono, 2007). The needle surfaces of *A. nebrodensis* trees most exposed to severe climatic conditions likely represent a less favorable microhabitat for fungal propagules due to lower humidity, high wind speed, and solar radiation (Gomes et al., 2018). Moreover, the absence of neighboring trees in Group C likely also contributes to lower diversity, as neighboring trees favor accumulation of fungal inoculum from diverse sporulating species (Ishii et al., 2004; Madi et al., 2020). As found by Kohler et al. (2025) and Inácio et al. (2002), neighborhood diversity promoted full phyllosphere fungal diversity without significantly affecting the endospheric community, which is more specialized and less influenced by external stimuli (Yao et al., 2019). Habitat effects also influenced differential abundance of specific groups: Valsaceae dominated in Group C trees under most extreme conditions, while Phaeosphaeriaceae and Dothideaceae showed higher frequencies in Group A.

While the present study did not incorporate quantitative habitat descriptors, someway limiting the understanding of the forces shaping *A. nebrodensis* mycobiome, this in part reflects the inherent complexity of characterizing natural environments, where a large proportion of the observed variation in associated microbiota often remains unexplained (Grilli, 2020; Trivedi et al., 2020). As also stated by these authors, such unexplained variation may arise from stochastic processes, unmeasured environmental factors, or fine-scale spatial heterogeneity that is difficult to quantify in complex forest settings. Moreover, although the collective influence of environmental conditions can strongly structure microbial communities, the effects of individual microhabitat variables are often subtle, interactive, and difficult to disentangle, particularly at fine spatial scales where multiple abiotic factors covary. Nevertheless, the pronounced compositional differences observed among habitat sites demonstrate that broad environmental conditions are strong and consistent drivers of the phyllosphere mycobiome structure of *A. nebrodensis*. Future studies incorporating fine-scale abiotic parameters measurements will build upon these findings and help disentangle the relative contributions of individual environmental variables to community variation.

### 4.5 Genotype effect

Despite evidence that host genotype shapes tree mycobiome composition (Elfstrand et al., 2020; Redondo et al., 2022), host genotype effect could not be assessed; previous evidence of low population diversity (Del Valle et al, 2025) is consistent with expected limited variation among trees, but this remains to be confirmed.

### 4.6 Core mycobiota

Plant-associated microorganisms not only play crucial roles in growth, development and physiology of their hosts, but also protect them from environmental stresses. The core microbiota may constitute dominant taxa carrying genes with central roles in plant growth, adaptation and defense (Lemanceau et al., 2017; Trivedi et al., 2020; Hawkes et al., 2021). Importantly, the identification of a stable core mycobiota has direct implications for the conservation of *A. nebrodensis*. Recent findings highlight that microbial communities can enhance the resilience of rare and threatened plants by improving stress tolerance and stabilizing plant performance under variable environmental conditions (reviewed by Crawford et al., 2026).

The phyllospheric core mycobiota of *A. nebrodensis* was represented by five classes: Dothideomycetes, Eurotiomycetes, Sordariomycetes, Leotiomycetes, and Lecanoromycetes. These were commonly found in the phyllosphere of conifers, broadleaves, shrubs, and other evergreen perennial plants, with Dothideomycetes often dominant (Lazarevic and Menkis, 2025; Botella et al., 2010; Macaya-Sanz et al., 2023; Ren et al., 2024; Costa et al., 2021). More ASVs were observed in the full phyllosphere core than the endospheric core (34 vs. 22), with Lecanoromycetes and three ASVs belonging to Botryosphaeriaceae, *Rhizosphaera*, and *Athallia* absent from the endospheric core. Botryosphaeriaceae are widespread on aerial woody plant parts, frequently acting as latent pathogens, endophytes, or saprobes (Slippers and Wingfield, 2007). Their absence from the endospheric core, consistent with the lack of cankers and diebacks in *A. nebrodensi*s, likely reflects competitive exclusion by better-adapted fungi.

In the endospheric community, H and S samples shared most families and genera, differing only in one *Lachnellula* ASV (symptomatic only) and one *Cladosporium* ASVs (healthy only). *Phaeosphaeria* was even more dominant in the endosphere, while Valsaceae were also highly represented, with 100% occurrence and 13% average abundance.

Valsaceae represented nearly one third of the total fungal community. Overall, 69 and 29 ASVs were assigned to this family in healthy and symptomatic samples, respectively. In both, just two ASVs accounted for 98% and 95% of total Valsaceae relative frequency, belonging to *Valsa friesii* and *Cytospora piceae*, respectively. These results are consistent with culture-based findings where *Valsa* and *Cytospora* were the most frequently isolated fungi from *A. nebrodensis*, detected in both symptomatic and asymptomatic needles (Frascella et al., 2024). The association of Valsaceae with firs across Europe has been reported in other studies (Lin et al., 2024; Lazarevic and Menkis, 2022). Their abundance in both tissue types and massive presence in the core mycobiota suggests a stable association with Sicilian fir. Although this cannot be proven by ITS2 metabarcoding, the results of this study, along with the high isolation rate observed in culture-based studies of both healthy and blighted needles (Frascella et al., 2024) is consistent with an important functional role of Valsaceae as transition fungi shifting from endophytism to saprophytism under host stress or needle senescence, a lifestyle transition documented in succession studies showing that most early colonizers and saprotrophs in leaf litter originate from endophytes (Sieber-Canavesi and Sieber, 1993; Promputtha et al., 2007; Ghimire and Hyde, 2004). Their greater abundance in Group C trees further supports this ecological function.

*Phaeosphaeria* is a Dothideomycetes found to be the dominant genus and main representative of the core mycobiota of *A. nebrodensis*. It was previously reported as dominant in marginal *A. alba* populations, in needles of *Pinus heldreichii* under harsh conditions, and as an endophyte in declining *P. halepensis* (Lazarevic and Menkis, 2025, 2020; Botella et al., 2010). Dothideomycetes can predominate fungal seed microbiome (Barret et al., 2015; Kinge et al., 2019) with *Phaeosphaeria* ranking among the most abundant genera, suggesting possible vertical transmission of this genus (Wassermann et al., 2019; Guo et al., 2021). This suggests that these fungi are widely spread among plants and may act as beneficial fungi, promoting seedling establishment, growth and development of host plants in nutrient-poor soils and under stressful conditions (War et al, 2023). Moreover, a series of microbial natural compounds were isolated from *Phaeosphaeria* fungi, exhibiting a broad spectrum of biological activities (El-Demerdash, 2018). It would be interesting to investigate whether and how *Phaeosphaeria* play a role in benefiting *A. nebrodensis* in its natural habitat through the production of secondary metabolites.

Other core genera including *Cladosporium*, *Perusta*, *Foliophoma*, *Alternaria*, and *Athallia* also belong to Dothideomycetes, which, as in several other studies, is the most represented class including fungi with a high level of ecological diversity (Traversari et al., 2025; Arnold et al., 2025; Macaya-Sanz et al., 2023; Costa et al., 2021; Lazarevic and Menkis, 2020).

We argue that the “extended plant phenotype” (Hawkes et al, 2021) encoded by the core microbiome represents a fundamental component of plant functioning and holobiont adaptation, particularly important in extreme environments. Noteworthy is the presence of five melanized genera (black fungi) in the core mycobiota, such as *Knufia*, *Perusta*, *Cladosporium*, *Alternaria*, and *Sarcinomyces*, all classified as extremotolerant and rock-inhabiting fungi. Melanin confers tolerance to UV radiation, desiccation, rehydration, and temperature fluctuations (Liu et al., 2022), all conditions characterizing the residual habitat of Sicilian fir. Enrichment of these melanized groups may reflect specific functional adaptation of the core mycobiota to promote holobiont survival in hostile conditions. However, the results of this study only allow us to suppose the possible role of these fungi, without being able to prove their function. An alternative explanation for their occurrence in the core mycobiota is that aerial deposition from adjacent rock surfaces accounts for their presence. Many genera of black fungi are known to behave as epiphytes or endophytes, e.g. the dark septate endophytes (DSE) residing in plant roots (Daghino et al., 2022). The close contact of lower branches with rocks in isolated *A. nebrodensis* trees likely facilitates horizontal transfer of rock-inhabiting propagules to leaf surfaces. The persistence of melanized fungi even in SS samples may also partly reflect the resistance of melanized mycelia to sterilization protocols (Isola et al., 2022).

*Alternaria* is cosmopolitan and highly adaptable fungus, exhibiting saprophytic, endophytic, opportunistic pathogenic, and aggressive parasitic behavior (DeMers, 2022). Endophytic strains may promote plant growth and stress resistance through secondary metabolites (Eram et al., 2018), while under stress conditions, endophytic or saprophytic strains may become pathogenic (Vinogradova et al., 2026). In our study, the same ASV of *Alternaria* was found in both healthy and symptomatic tissues, suggesting the capacity to shift relationships with the host. *Alternaria* communities have been reported to exhibit a high activity in producing leaf decomposition enzymes, thus contributing to nutrient cycling (Zhang et al., 2024). *Cladosporium*, another cosmopolitan genus with over 700 species, comprises common endophytes (Riesen and Sieber 1985; Brown et al., 1998; El-Morsy, 2004), secondary invaders, and phylloplane fungi (Islam and Hasin, 2000; de Jager et al., 2001; Inácio et al., 2002, Stohr and Dighton 2004; Levetin and Dorseys 2006), producing diverse bioactive secondary metabolites including antifungal and antibacterial compounds, and serving as a biocontrol agent. Its presence in both healthy and blighted core endospheric samples likely reflects an endophytic to saprophytic behavioral shift. *Perusta* is a genus of extremotolerant fungi with a specific, recently described ecology, predominantly associated with rock-inhabiting environments and endophytic roles (Liu et al., 2022). It is known as an early-stage colonizer in larch (*Larix gmelinii*) leaf litter decomposition (Pan et al., 2024), explaining its core presence in both healthy and symptomatic samples of *A. nebrodensis* in this study. *Knufia* (Chaetothyriales) comprises melanized, slow-growing fungi acting as rock-inhabiting extremotolerant species and dark septate endophytes (DSE). Inoculation with *Knufia* sp. can increase antioxidant enzyme activity and improve host water stress tolerance (Li et al., 2019), suggesting that this stable association may play an important role for *A. nebrodensis* adaptation. Similarly, *Sarcinomyces* possess thick melanized cell walls and colonizes rocky outdoor surfaces; *S. crustaceus* has been isolated as an endophyte from *Quercus* internal tissues (Novotny, 2022).

Among the other genera being part of the core mycobiota of *A. nebrodensis*, *Lachnellula*, includes pathogenic or opportunistic species causing cankers on twigs, branches and stems of conifers subjected to stresses or living in sub-optimal sites (Oguchi, 1979; Smerlis, 1973). It has also been reported as saprophyte in decomposing wood, fallen leaves and litter (Filimonova et al., 2025) and as an endophyte (Lazarević and Menkis, 2020; Moler and Aho, 2018; Park et al., 2018). Its occurrence uniquely in the core of symptomatic samples, suggests an opportunistic-saprophytic role in *A. nebrodensis* phyllosphere. *Foliophoma* (Pleosporales, Dothideomycetes) is also part of the core mycobiota of *A. nebrodensis* and has been reported among the most abundant fungi in olive leaves affected by leaf spot and is a component of olive endophytic core microbiota (Varanda et al., 2019; Gomes et al., 2023; Costa et al., 2021). Some *Foliophoma* species are known to produce potent antifungal compounds (Crespo et al., 2020). *Phaeomoniella*, primarily known for causing Petri disease and Esca in grapevines but also affecting other woody hosts as a latent pathogen (Hrycan et al., 2020), likely plays a role as an early decomposer in the *A. nebrodensis* ecosystem.

### 4.7 Functional roles

To propose hypotheses about the functional ecology of the taxa associated with the *A. nebrodensis* phyllosphere, we performed putative “guilds” and “lifestyles” annotation using FUNGuild and FungalTraits, respectively: overall, the analysis suggested that the phyllosphere of Sicilian fir is dominated by fungal taxa reported in the literature as endophyte- and saprotroph-associated. The occurrence of taxa reported as plant pathogens in both healthy and symptomatic samples indicates that the mere presence of potentially pathogenic fungi is insufficient to explain symptom development. The plant pathogen lifestyle putative annotation via FungalTraits consistently with FUNGuild provides not conclusive evidence of active fungal pathogenesis in symptomatic *A. nebrodensis*.

Previous study from larch needle (Pan et al., 2024) shows that many fungi can persist as endophytes in living tissues and later shift toward saprotrophic or opportunistic pathogenic activity following senescence or stress. This framework provides a plausible explanation for the high proportion of taxa annotated as endophytes in A. nebrodensis, but alternative functional roles cannot be excluded.

Interestingly, the higher representation of lichenized fungi in non-sterilized samples may reflect epiphytic colonization of needle surfaces and highlights the ecological complexity of the Sicilian fir phyllosphere habitat.

However, these ecological interpretations should be regarded as hypothesis-generating rather than definitive, and future studies integrating functional, genomic, or transcriptomic approaches will be necessary to validate the ecological roles of the various taxa presented.

## 5 Conclusions

This study analyzes for the first time the fungal community of the phyllosphere of the critically endangered *A. nebrodensis* using HTS metabarcoding techniques. Health state of tissues and compartment (needle/twig) do not always display a significant effect in the variation of the phyllosphere mycoflora.

The main factor shaping the phyllosphere fungal assemblage is the site-specific variation in environmental conditions within the habitat of *A. nebrodensis*. Isolated trees, close to ridges with shallow, rocky soils, exposed to strong winds and intense sunlight, showed a greater abundance of Valsaceae, the dominant family and part of the core mycobiota of the Sicilian fir. The taxa dominating the phyllosphere fungal communities of *A. nebrodensis* can be classified as endophytes, weak pathogens and facultative saprotrophs. This outcome, along with the absence of primary pathogens, confirms that shoot and needle blight symptoms observed on the crowns are triggered by environmental stresses, as reported in previous studies.

The core mycobiota consists of fungal taxa that appear to play a functional role in promoting fitness of the host to the hard conditions of the habitat in which it resides. Valsaceae, also abundantly found in both healthy and symptomatic needles in previous culture-based study on *A. nebrodensis*, may play a role as transition fungi that initiate the process of senescence and decomposition of needles and twigs and may be therefore useful to the nutrient cycle. The participation of five genera of melanized and extremotolerant fungi (*Knufia*, *Perusta*, *Sarcinomyces*, *Alternaria*, *Cladosporium*) in the core mycobiota is likely driven by the harsh habitat. The possibility that these fungi play a functional role in promoting the holobiont’s adaptation to a hostile environment, thereby contributing to the host’s tolerance to environmental stresses, is intriguing and warrants further investigation. Other dominant fungi such as *Phaeosphaeria*, *Lachnellula, Foliophoma*, and *Phaemoniella* may play a role in the nutrient cycle, as weak pathogens and saprophytes and/or as beneficial fungi thanks to the production of secondary metabolites.

Further studies should focus on characterizing the interaction between dominant fungi and the host, also with a view to identifying beneficial microorganisms that can be deployed to promote growth and stress tolerance of plants in reforestation programs. Both *in situ* and *ex situ* conservation strategies for endangered plants may benefit from the safeguard of the microbiota associated to the host plant.

The high fungal diversity detected in the phyllosphere of *A. nebrodensis*, suggests this relic species is a reservoir of microbial diversity that is relevant in its adaptation and highlights the ecological role of phyllosphere fungi in forest ecosystems. Efforts to maximize biodiversity conservation, generally aimed at counteracting habitat degradation processes driven by climate and human activities, cannot overlook preserving microbial diversity which is essential for ecosystem functions.

## Data Availability

The datasets presented in this study can be found in online repositories. The names of the repository/repositories and accession number(s) can be found below: https://www.ncbi.nlm.nih.gov/genbank/, Bioproject PRJNA1097375.
