# Supplementary material for: Habitat conditions shape the phyllosphere mycobiome of the critically endangered Abies nebrodensis
Source: Front Plant Sci. 2026 Jul 24;17:1876158. doi: 10.3389/fpls.2026.1876158 (PMC13447281; doi:10.3389/fpls.2026.1876158)
Supplement: Supplementary file 1 [file Table1.docx]

Supplementary Material

# 1 Supplementary Figures and Tables

## 1.1 Supplementary Figures

**Supplementary Figure 1.** Per sample accumulation curves of AVS in relation to sequencing depth

**Supplementary Figure 2**. Tax prevalence plot of the full *A. nebrodensis* mycobiome

**Supplementary** **Figure 3.** Venn diagram of the ASVs building the complete phyllosphere (NS) or strictly endospheric (SS) microbiota.

**Supplementary Figure 4.** Alpha diversity indices of the phyllosphere endophytic fungal communities: **(A)** organ specific differences (needle/twig); **(B)** health status (healthy/symptomatic); **(C)** cluster of plants (A/B/C). * *p* < 0.05, ** *p* < 0.01, *** *p* < 0.001, **** *p* < 0.0001

**Supplementary Figure 5.** Bray-Curtis non phylogenetic beta-diversity of the phyllosphere endophytic fungal communities: top left, organ specific differences (needle/twig); top right, health status (healthy/symptomatic); bottom left, cluster of trees (A/B/C); bottom right, Permanova with 999 permutations.

**Supplementary Figure 6.** Differential abundance of the top taxonomically resolved families in NS and SS samples groups

**Supplementary Figure 7**. Differential abundance of the top genus in NS and SS samples groups; this plot shows also taxonomically unresolved groups at genus level.

**Supplementary Figure 8.** Differential abundance of the top abundant taxonomically resolved families of the full fungal community (NS) in relation to the health status (healthy/symptomatic).

**Supplementary Figure 9.** Differential abundance of the top abundant taxonomically resolved genera of the full fungal community (NS) in relation to the health status (healthy (H)/symptomatic (S)).

**Supplementary Figure 10.** Differential abundance of the top taxonomically resolved genus in NS and SS samples groups.

**Supplementary Figure 11.** Differential abundance of the top abundant taxonomically resolved families of the endospheric fungal community (SS) in relation to the health status (healthy (H)/symptomatic (S)).

**Supplementary Figure 12**. Differential abundance of the top abundant taxonomically resolved genera of the endospheric fungal community (SS) in relation to the health status (healthy (H)/symptomatic (S)).

**Supplementary Figure 13A**. Taxa bar plot depicting the composition (relative abundance) of the full (NS) or strictly endospheric (SS) communities of symptomatic samples. Only the 9 most abundant families (on the left) or genera (on the right) are reported. The group “other” includes all remaining families (or genera).

**Supplementary Figure 13B.** Differential abundance of the top abundant taxonomically resolved families of the full (NS) or endospheric (SS) mycobiota of the symptomatic samples.

**Supplementary Figure 14.** Differential abundance of the top abundant genera of the phyllosphere fungal community (NS) in relation to the ecological context of vegetation (tree clusters A, B, and C)

**Supplementary figure 15.** Taxa bar plot depicting the composition (relative abundance) of the endospheric communities of samples from the tree clusters A, B, and C. Only the 9 most abundant families (on the left) or genera (on the right) are reported, a category “other” sums all remaining families (or genera)

**Supplementary Figure 16.** Putative functional assignments of ASVs to primary lifestyle categories using FungalTraits (Põlme et al., 2020). These assignments are trait-based predictions inferred from taxonomic identity and have not been experimentally validated. Results are shown for the *Abies nebrodensis* phyllosphere across three sites (A, B, C) in Healthy and Symptomatic samples, performed separately for A) NS samples (full phyllosphere) and B) SS samples (endosphere).

## 1.2 Supplementary Tables

**Supplementary Table 1**. Permanova analysis (999 permutations), considering the surface sterilization process (treatment) as the variable.

|  | Df | SumOfSqs | R^2^ | F | Pr(>F) |
| --- | --- | --- | --- | --- | --- |
| Treatment | 1 | 1.21412 | 0.05812662 | 4.56682368 | 0.002 |
| Residual | 74 | 19.6733849 | 0.94187338 | NA | NA |
| Total | 76 | 20.8875049 | 1 | NA | NA |

**Supplementary Table 2.** Composition of the endophytic core fungal microbiota in healthy samples. ASVs in bold are unique to the core microbiota of healthy samples. Only ASVs annotated at least at family level are reported.

| ASV | Phylum | Class | Order | Family | Genus |
| --- | --- | --- | --- | --- | --- |
| 73cd44c9009aa96d3e0b57b8b79ab23b | **Ascomycota** | **Dothideomycetes** | **Capnodiales** | **Cladosporiaceae** | **Cladosporium** |
| e612009ed4ba84e81015803afccb2fb3 | Ascomycota | Dothideomycetes | Capnodiales | Cladosporiaceae | Cladosporium |
| 0f99029ba920469b3749e683b8d52a5d | Ascomycota | Eurotiomycetes | Chaetothyriales | Trichomeriaceae | Knufia |
| 545c0066f65f696759efec70e7120b4b | Ascomycota | Eurotiomycetes | Chaetothyriales | Chaetothyriales_fam_Incertae_sedis | Sarcinomyces |
| 62ebf8c0bc7886f0b0d497633696a264 | Ascomycota | Sordariomycetes | Diaporthales | Valsaceae |  |
| f60b0e31ff91e3a97b2c1b26d680caa5 | Ascomycota | Sordariomycetes | Diaporthales | Valsaceae |  |
| 160768f793322df7b8e49a42a41828fc | **Ascomycota** | **Dothideomycetes** | **Dothideales** | **Dothioraceae** | **Perusta** |
| 1a54f3540bc3733f3a0e6d6d32d29da9 | Ascomycota | Dothideomycetes | Dothideales | Dothioraceae | Perusta |
| 365cfa8cd5cfa1fa6e2fa76b9ff2266a | Ascomycota | Dothideomycetes | Dothideales | Dothioraceae | Perusta |
| 5d8cf88ffc717202e33288bb13599d90 | Ascomycota | Eurotiomycetes | Phaeomoniellales | Phaeomoniellaceae | Phaeomoniella |
| a2b5ca861d48b40515cc6b393d11e5a8 | Ascomycota | Dothideomycetes | Pleosporales | Coniothyriaceae | Foliophoma |
| 25db62c6ae2219d813729b24dbc5967b | Ascomycota | Dothideomycetes | Pleosporales | Pleosporaceae | Alternaria |
| c5c87ba4d87feba44245ea5d32103809 | **Ascomycota** | **Dothideomycetes** | **Pleosporales** | **Didymellaceae** |  |
| 1c8c97fe5f9061636e16ea35de915966 | Ascomycota | Dothideomycetes | Pleosporales | Phaeosphaeriaceae | Phaeosphaeria |
| 3df1eaa89f96ded1dcc7eccb7ebc7f61 | Ascomycota | Dothideomycetes | Pleosporales | Phaeosphaeriaceae | Phaeosphaeria |
| a323b0fe2e79b8de3fecabd0545f59ba | Ascomycota | Dothideomycetes | Pleosporales | Phaeosphaeriaceae | Phaeosphaeria |
| f1480e3b712a22b242082b9b3125919b | Ascomycota | Sordariomycetes | Xylariales | Sporocadaceae |  |

**Supplementary Table 3.** Composition of the endophytic core fungal microbiota in symptomatic samples. ASVs in bold are unique to the core microbiota of symptomatic samples. Only ASVs annotated at least at family level are reported

| ASV | Phylum | Class | Order | Family | Genus |
| --- | --- | --- | --- | --- | --- |
| e612009ed4ba84e81015803afccb2fb3 | Ascomycota | Dothideomycetes | Capnodiales | Cladosporiaceae | Cladosporium |
| 0f99029ba920469b3749e683b8d52a5d | Ascomycota | Eurotiomycetes | Chaetothyriales | Trichomeriaceae | Knufia |
| 545c0066f65f696759efec70e7120b4b | Ascomycota | Eurotiomycetes | Chaetothyriales | Chaetothyriales_fam_Incertae_sedis | Sarcinomyces |
| 62ebf8c0bc7886f0b0d497633696a264 | Ascomycota | Sordariomycetes | Diaporthales | Valsaceae |  |
| f60b0e31ff91e3a97b2c1b26d680caa5 | Ascomycota | Sordariomycetes | Diaporthales | Valsaceae |  |
| 1a54f3540bc3733f3a0e6d6d32d29da9 | Ascomycota | Dothideomycetes | Dothideales | Dothioraceae | Perusta |
| 365cfa8cd5cfa1fa6e2fa76b9ff2266a | Ascomycota | Dothideomycetes | Dothideales | Dothioraceae | Perusta |
| f7e61533bd27a510d9217348a8837af3 | **Ascomycota** | **Leotiomycetes** | **Helotiales** | **Hyaloscyphaceae** | **Lachnellula** |
| 5d8cf88ffc717202e33288bb13599d90 | Ascomycota | Eurotiomycetes | Phaeomoniellales | Phaeomoniellaceae | Phaeomoniella |
| 27bd9c188bfc401eeffeccfe504e552f | **Ascomycota** | **Dothideomycetes** | **Pleosporales** | **Coniothyriaceae** | **Foliophoma** |
| a2b5ca861d48b40515cc6b393d11e5a8 | Ascomycota | Dothideomycetes | Pleosporales | Coniothyriaceae | Foliophoma |
| 25db62c6ae2219d813729b24dbc5967b | Ascomycota | Dothideomycetes | Pleosporales | Pleosporaceae | Alternaria |
| 1c8c97fe5f9061636e16ea35de915966 | Ascomycota | Dothideomycetes | Pleosporales | Phaeosphaeriaceae | Phaeosphaeria |
| 3df1eaa89f96ded1dcc7eccb7ebc7f61 | Ascomycota | Dothideomycetes | Pleosporales | Phaeosphaeriaceae | Phaeosphaeria |
| a323b0fe2e79b8de3fecabd0545f59ba | Ascomycota | Dothideomycetes | Pleosporales | Phaeosphaeriaceae | Phaeosphaeria |
| f1480e3b712a22b242082b9b3125919b | Ascomycota | Sordariomycetes | Xylariales | Sporocadaceae |  |

**Supplementary Table 4.** Relative abundance of the family Valsaceae in relation to the tree clusters (A, B or C) and the phytosanitary status (H = healthy, S = symptomatic).

| **Cluster** | **Health** | **Abundance (Mean ± SD)** |
| --- | --- | --- |
| A | S | 0.087 ± 0.115 |
| A | H | 0.223 ± 0.348 |
| B | S | 0.336 ± 0.381 |
| B | H | 0.129 ± 0.078 |
| C | S | 0.345 ± 0.362 |
| C | H | 0.399 ± 0.436 |

**Supplementary Table 5.** Full ASVs naming and recoding correspondence for the core microbiomes. NSX = only in the full phyllospheric core, SSX=only in the endospheric microbiome, SHX = shared. Only ASV annotated at least at Order level are reported.

| Original ID | New code | Label | Group |
| --- | --- | --- | --- |
| **Full phyllospheric (NS) core microbiome** | | | |
| 62ebf8c0bc7886f0b0d497633696a264 | SH1 | SH1 - Valsaceae | shared |
| f60b0e31ff91e3a97b2c1b26d680caa5 | SH2 | SH2 - Valsaceae | shared |
| 47b7b208c21dfe0bcead7ef7a6531696 | NS1 | NS1 – Knufia aspidiotus | NS |
| 0f99029ba920469b3749e683b8d52a5d | SH3 | SH3 – Knufia aspidiotus | shared |
| 5d8cf88ffc717202e33288bb13599d90 | SH4 | SH4 - Phaeomoniella | shared |
| 3e075afd642b26b216e96a975d641bba | NS2 | NS2 - Dothideomycetes | NS |
| 97a1e64b4604d870d0236a6d8506917f | NS3 | NS3 - Dothideomycetes | NS |
| d888aabd772e8a562dabacca76774b69 | SH5 | SH5 - Coryneliales | shared |
| f11a794a4dc518da6a32ca9b44933892 | SH6 | SH6 - Coryneliales | shared |
| 27bd9c188bfc401eeffeccfe504e552f | NS4 | NS4 – Foliophoma camporesii | NS |
| a2b5ca861d48b40515cc6b393d11e5a8 | SH7 | SH7 – Foliophoma camporesii | shared |
| 726fc9bf966a2baf9dd21fe621d49bf5 | SH8 | SH8 - Pleosporales | shared |
| a323b0fe2e79b8de3fecabd0545f59ba | SH9 | SH9 - Phaeosphaeria | shared |
| ebf583f9ea420719f7cecf59483af2ea | NS5 | NS5 - Rhizosphaera | NS |
| 999cf59caa922fe2ef43879fb35b232c | SH10 | SH10 - Dothideales | shared |
| 1a54f3540bc3733f3a0e6d6d32d29da9 | SH11 | SH11 - Perusta | shared |
| 365cfa8cd5cfa1fa6e2fa76b9ff2266a | SH12 | SH12 - Perusta | shared |
| 545c0066f65f696759efec70e7120b4b | SH13 | SH13 – Sarcinomyces crustaceus | shared |
| 73cd44c9009aa96d3e0b57b8b79ab23b | SH14 | SH14 – Cladosporium herbarum | shared |
| e612009ed4ba84e81015803afccb2fb3 | SH15 | SH15 - Cladosporium | shared |
| ca2b9c7bf3fe4d51c7067d81ed2bd42b | NS6 | NS6 - Dothideomycetes | NS |
| 2b93da215da31eafaaeafe4373271463 | NS7 | NS7 - Athallia_alnetorum | NS |
| f7e61533bd27a510d9217348a8837af3 | SH16 | SH16 – Lachnellula calyciformis | shared |
| b98c8d1cc295f655fd640d903592fbb3 | NS8 | NS8 - Dothideomycetes | NS |
| a1c19db26af1266fb64f27279874e618 | NS9 | NS9 - Dothideomycetes | NS |
| 962afe5b65eae495322c8d06d67bec70 | NS10 | NS10 - Botryosphaeriaceae | NS |
| **Endospheric (SS) core microbiome** | | | |
| 62ebf8c0bc7886f0b0d497633696a264 | SH1 | SH1 - Valsaceae | shared |
| f60b0e31ff91e3a97b2c1b26d680caa5 | SH2 | SH2 - Valsaceae | shared |
| f1480e3b712a22b242082b9b3125919b | SS1 | SS1 - Sporocadaceae | SS |
| 0f99029ba920469b3749e683b8d52a5d | SH3 | SH3 – Knufia aspidiotus | shared |
| 5d8cf88ffc717202e33288bb13599d90 | SH4 | SH4 - Phaeomoniella | shared |
| d888aabd772e8a562dabacca76774b69 | SH5 | SH5 - Coryneliales | shared |
| f11a794a4dc518da6a32ca9b44933892 | SH6 | SH6 - Coryneliales | shared |
| a2b5ca861d48b40515cc6b393d11e5a8 | SH7 | SH7 – Foliophoma camporesii | shared |
| 25db62c6ae2219d813729b24dbc5967b | SS2 | SS2 – Alternaria alternata | SS |
| 726fc9bf966a2baf9dd21fe621d49bf5 | SH8 | SH8 - Pleosporales | shared |
| 1c8c97fe5f9061636e16ea35de915966 | SS3 | SS3 - Phaeosphaeria | SS |
| 3df1eaa89f96ded1dcc7eccb7ebc7f61 | SS4 | SS4 - Phaeosphaeria | SS |
| a323b0fe2e79b8de3fecabd0545f59ba | SH9 | SH9 - Phaeosphaeria | shared |
| 999cf59caa922fe2ef43879fb35b232c | SH10 | SH10 - Dothideales | shared |
| 160768f793322df7b8e49a42a41828fc | SS5 | SS5 - Perusta | SS |
| 1a54f3540bc3733f3a0e6d6d32d29da9 | SH11 | SH11 - Perusta | shared |
| 365cfa8cd5cfa1fa6e2fa76b9ff2266a | SH12 | SH12 - Perusta | shared |
| 545c0066f65f696759efec70e7120b4b | SH13 | SH13 – Sarcinomyces crustaceus | shared |
| 73cd44c9009aa96d3e0b57b8b79ab23b | SH14 | SH14 – Cladosporium herbarum | shared |
| e612009ed4ba84e81015803afccb2fb3 | SH15 | SH15 - Cladosporium | shared |
| f7e61533bd27a510d9217348a8837af3 | SH16 | SH16 – Lachnellula calyciformis | shared |
